# First do no harm: pain relief for the peripheral venous cannulation of adults, a systematic review and network meta-analysis

**DOI:** 10.1186/s12871-016-0252-8

**Published:** 2016-10-01

**Authors:** Mary Bond, Louise Crathorne, Jaime Peters, Helen Coelho, Marcela Haasova, Chris Cooper, Quentin Milner, Vicki Shawyer, Christopher Hyde, Roy Powell

**Affiliations:** 1 University of Exeter Medical School, University of Exeter, Veysey Building, Salmon Pool Lane, Exeter, EX2 4SG UK; 2 Department of Anaesthesia, Royal Devon and Exeter Foundation Trust, Exeter, UK; 3 Vascular Access team, Royal Devon and Exeter Foundation Trust, Exeter, UK; 4 Research Design Service South West, Exeter, UK

**Keywords:** Adult, Peripheral venous catheterization, Pain, Local anaesthetic, Network meta-analysis, Systematic review

## Abstract

**Background:**

Peripheral venous cannulation is an everyday practice in hospitals, which many adults find painful. However, anaesthesia for cannulation is usually only offered to children. Inadequate pain relief is not only unpleasant for patients but may cause anxiety about further treatment and deter patients from seeking medical care in the future. The aim of this study is to discover the most effective local anaesthetic for adult peripheral venous cannulation and to find out how the pain of local anaesthetic application compares with that of unattenuated cannulation.

**Methods:**

These aims are addressed through a systematic review, network meta-analysis and random-effects meta-analysis. Searching covered 12 databases including MEDLINE and EMBASE from 1990 to August 2015. The main included study design was RCTs. The primary outcome measure is self-reported pain, measured on a 100 mm visual analogue scale.

**Results:**

The systematic review found 37 includable studies, 27 of which were suitable for network meta-analysis and two for random-effects meta-analysis. The results of the network meta-analysis indicate that none of the 17 anaesthetic considered had a very high probability of being the most effective when compared to each other; 2 % lidocaine had the highest probability (44 %). When the anaesthetics were compared to no treatment, the network meta-analysis showed that again 2 % lidocaine was estimated to be the most effective (mean difference −25.42 (95 % CI −32.25, −18.57). Other members of the ‘caine’ family were also estimated to be more effective than no treatment as were Ametop^®^, EMLA^®^ and Rapydan^®^ patch. The meta-analysis compared the pain of anaesthetic application with the unattenuated pain of cannulation. This found that all applications of local anaesthetic were less painful than cannulation without local anaesthetic. In particular a 1 % lidocaine injection was estimated to be −12.97 (95 % CI −15.71, −10.24) points (100 mm VAS) less painful than unattenuated cannulation.

**Conclusions:**

The pain of peripheral venous cannulation in adults can be successfully treated. The pain of application of any local anaesthetic is less than that of unattenuated cannulation. Local anaesthetic prior to cannulation should become normal practice and a marker of high quality care.

**Protocol registration:**

The protocol for the larger study was registered with PROSPERO no. CRD42012002093.

**Electronic supplementary material:**

The online version of this article (doi:10.1186/s12871-016-0252-8) contains supplementary material, which is available to authorized users.

## Background

First do no harm. It can be argued that causing unnecessary pain during medical procedures is doing harm. One example is the routine insertion of peripheral venous cannulae (PVC). This procedure is a common experience for thousands of patients and reported by adults to be painful [1]. Although it is normal practice to provide local anaesthesia for children prior to PVC it is unusual for this to be offered to adults.

Inadequate pain relief is unpleasant for the patient but may also increase anxiety about future treatment and deter patients from seeking help in the future [2–5]. Fear of the procedure can trigger an autonomic response, which can result in vasoconstriction [6, 7]. This has the potential to cause reduced venous access, potentially making PVC more difficult for the practitioner leading to several attempts at insertion, thus increasing the risk of infection and other complications [8–10].

A survey investigating the use of local anaesthesia for adult PVC questioned 178 hospital doctors. It found that all the anaesthetists used local anaesthetic when inserting cannulae larger than 18 gauge, but less than half medical and surgical doctors did so [11]. Another survey of 71 junior doctors’ use of and attitudes to pain relief for adult PVC gave some reasons for this discrepancy. It showed that 35 % of junior doctors sometimes used a local anaesthetic. However, those using local anaesthetic only did so on average for 6 % of the time. Most of these used injected lidocaine (84 %) others used eutectic mixture local anaesthetics (EMLA^®^) cream (48 %) with 36 % using either agent. The 65 % who never used a local anaesthetic for PVC in adults gave a variety of reasons for this, including that it was, too time consuming (45 %), not indicated (35 %), made PVC more difficult (21 %), not available (13 %), logistically difficult (13 %), against peer pressure (4 %), not allowed (4 %) and practically difficult (4 %) [12].

Although trials have been conducted comparing various local anaesthetic agents for adult PVC [13–17] and there have been two meta-analyses in the adult population, one of lidocaine and one of EMLA^®^ [18, 19] it remains unknown which of the many agents in use is the most effective. This paper represents some of the findings of a larger systematic review, which aimed to address this knowledge gap [20]. This larger report is available on request from the authors.

## Methods

### Aim

The aim of this research paper is to answer the following research questions:

1. What is the most effective local anaesthetic for reducing the pain of PVC in adults in routine (non-emergency) settings?
2. How does the pain of local anaesthetic application compare with that of routine (non-emergency) unattenuated PVC in adults?

We answered these questions by conducting a systematic review, network meta-analysis and random-effects meta-analysis.

The systematic review was carried out following the principles published by the National Health Service (NHS) Centre for Reviews and Dissemination [21].

### Eligibility criteria

Eligible studies included both controlled trials and observational studies that compared the use of a local anaesthetic prior to PVC with no local anaesthetic prior to PVC in adults in secondary care receiving routine PVC (non-emergency). The primary outcome measure was self-reported pain.

### Search strategy

The search strategy was developed by a professional Information Specialist (CC) and is provided in Additional file 1. The database searches were conducted in March 2012 and updated in June 2013, September 2014 and August 2015 using a protocol driven search. The following bibliographic resources were searched: MEDLINE, MEDLINE-IN-Process, EMBASE, PsycINFO, Health Management Information Consortium (HMIC), Social Policy and Practice (all via OVID), Applied Social Sciences Index and Abstracts (ASSIA), Sociological Abstracts (via ProQuest), Cumulative Index to Nursing and Allied Health Literature (CINAHL) (via Ebsco Host), British Nursing Index (via NHS Evidence), Web of Science (via Thomson Reuters) and the Cochrane library. Database searching was limited by date (1990-Current) and to human only populations. No further limits were used.

Web and grey literature searching was conducted using both Google and the meta-search engine Dogpile. The following web-sites were also searched: The Patients Association, NHS Evidence, National Institute for Health and Care Excellence (NICE), Current Controlled Trials, and Clinical Trials.gov. Citation chasing and contact with experts was used on publications included in the searches.

### Study selection, data extraction and quality assessment

Titles and abstracts were screened independently by three researchers (LC, MB and HC) against the inclusion criteria. Papers selected for full-text review underwent the same process. Data were extracted from included studies by one reviewer and checked by another. Study authors were contacted as necessary.

The risk of bias was assessed using the appropriate tool for the design of the study; the CONSORT (Consolidated Standards of Reporting Trials) statement [22] for randomised controlled trials (RCTs) and controlled trials, and the STROBE (STrengthening the Reporting of OBservational studies in Epidemiology) statement [23] for observational studies. External validity was judged according to the applicability of findings to the relevant patient group and service setting.

### Statistical analysis

The heterogeneity of RCTs was initially explored by assessing study population, methods and interventions. The principal summary measure was mean differences in self-reported pain. This was assumed to be linear on either 0–10 or 0–100 scales. To assist with the interpretation of the results, all responses on the scales were linearly rescaled to a range of 0–100. Thus, a mean difference between groups of −10 would indicate that post-intervention pain was 10 units lower in the intervention group compared with the control group. As some studies used medians to summarise their results, to allow analysis we transformed all estimates to means and standard deviations [24]. To address the first question a network meta-analysis (NMA) was undertaken to compare multiple treatments directly and indirectly, within and across trials. A model with normal likelihood and an identity link was used. All prior distributions were intended to be vague. Both a fixed effects and a random effects NMA model were run, but as the deviance information criteria suggested a better fit to the random effects model, only the results from the random effects model are reported here [25]. An analysis of the consistency of estimates derived from the direct and indirect evidence was also done [26]. The NMA was undertaken in WinBUGS. Analyses were run with three chains, and model convergence was assessed by visual inspection of density and autocorrelation plots, and checking that all three chains were sampling from the same posterior distribution.

As only two studies were eligible to answer the second question a fixed effects pairwise meta-analysis was undertaken in Stata SE 12 (Texas USA). Statistical heterogeneity was assessed by the I^2^ statistic. Formal evaluation of the risk of publication bias was not conducted for those studies included in addressing question one due to a lack of methods for assessing the risk of publication bias in NMAs. As there were only two studies eligible to answer Question 2, formal evaluation of the risk of publication bias was not conducted.

## Results

**Fig. 1:**
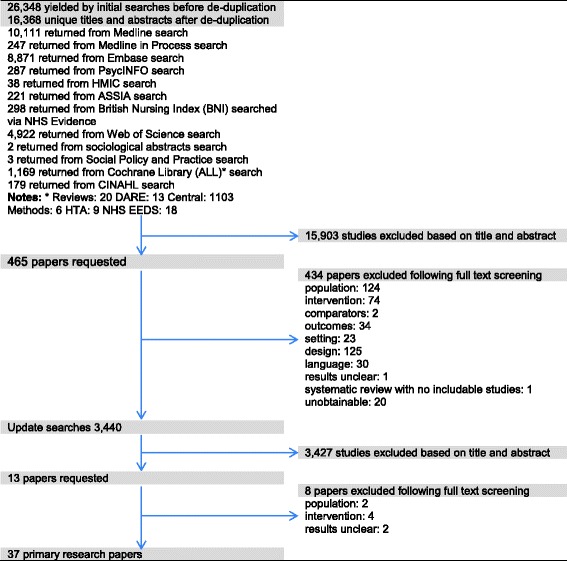
Flowchart of studies through the screening process

The initial searches found 16,368 titles and abstracts after deduplication. Following screening 465 papers were requested for further review; of these 20 were not obtainable. Of the 445 papers obtained 31 were found to meet the inclusion criteria. Reasons for exclusion can be found in Fig. 1. Update searches (September 2014 and August 2015) identified six additional studies, giving a total of 37 primary research studies. Thirty two were randomised controlled trials [1, 13–17, 27–52], four were controlled trials [53–56]} and one was a survey [57].

The primary outcome, pain, was measured in a variety of ways including: a visual analogue scale (VAS) (*n* = 22 studies [1, 14–17, 27, 30–32, 35, 37, 39–41, 43, 44, 46–49, 51–53, 56]), a numerical rating scale (*n* = 6, [13, 29, 34, 38, 50, 54]), Wong Baker Faces (*n* = 1, [28]) and, other (*n* = 4 [30, 33, 36, 45]). Four studies reported that participants had been given pre-medication prior to PVC [29, 42, 45, 47]. Cannulae were placed in the following sites: the dorsum of the hand (*n* = 27 [1, 13–17, 27, 29, 31, 32, 34–52, 54, 55]), the forearm (*n* = 12 [13, 28, 31, 33, 40, 49–52, 55, 56]), the wrist (*n* = 4 [31, 39, 40, 55]), and the anticubital fossa (*n* = 5 [13, 31, 39, 40, 52]). The location was not reported in three studies. The gauge of the cannulae ranged from 16 to 23, with 15 studies using an 18 gauge cannula [13, 14, 17, 27, 29, 31, 33, 34, 37, 39, 41–43, 46–48, 55], seven using a 20 gauge cannula [1, 13, 15, 30, 32, 44, 49, 54], and five studies not reporting the size of the cannula. A description of the local anaesthetics used in the included studies can be found in Additional file 2. Note than iontocaine is a compound containing 2 % lidocaine. A summary of the studies characteristics can be found in Additional file 3.

### Quality of evidence

The included studies varied in the quality of their reporting of the risk of bias. Type of randomisation, methods used to generate the random sequence, allocation concealment, and blinding were often not reported fully or at all. However, the statistical methods used for the primary analysis were clear and the estimates of mean differences with precision were generally reported. Additionally, the studies varied in size, ranging from 26 [39] to 450 [42] participants. Additional files 4–5 provide a summary critical appraisal of included studies.

### Study results

A summary of results from the included studies can be found in Additional file 6.

#### Question 1. What is the most effective local anaesthetic for reducing the pain of PVC in adults in routine settings?

**Fig. 2:**
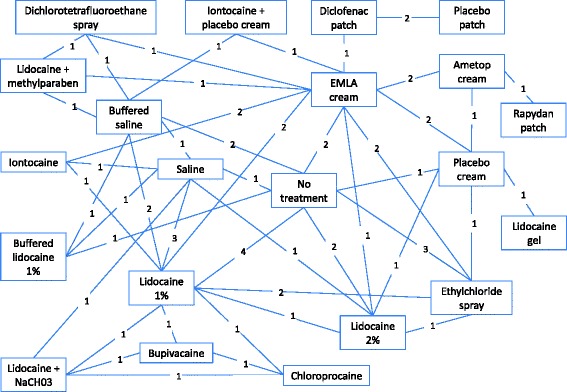
Network diagram

The NMA included 27 RCTs [1, 13, 15, 17, 27–38, 40, 41, 43–46, 48–52]. The other five RCTs were not included in the NMA because four did not provide enough information to be able to calculate standard deviations when transforming medians to means (Saxena [14], Agarwal [16, 42], Gupta [47]), and one concerned duration of anaesthetic application [39]. The included studies evaluated the following local anaesthetics: lidocaine 2 %, lidocaine 1 %, buffered lidocaine 1 %, lidocaine + methylparaben, lidocaine + NaCHO3, iontocaine, bupivacaine, Rapydan^®^, EMLA^®^, Ametop^®^, buffered saline, ethyl chloride, chloroprocaine, dichlorotetrafluoroethane, diclofenac, saline, placebo and no treatment. The network of included studies can be seen in Fig. 2. Note that many of the comparisons are just informed by one or two studies.

None of the treatments had a high probability of being the most effective compared to all of the treatments evaluated in the network. Two percent lidocaine had the highest probability of being the most effective treatment (44 %), followed by lidocaine + methylparaben (17 %) and iontocaine (15 %). Most treatments had a probability <1 % of being the most effective treatment, this included EMLA^®^, 1 % lidocaine, and Ametop^®^. The order of the rankings can be found in Additional file 7.

**Fig. 3:**
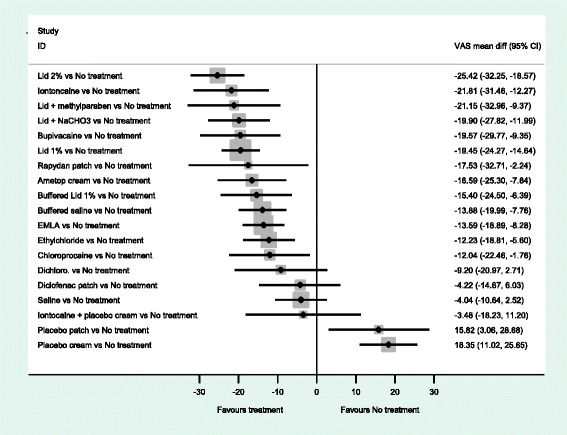
NMA forest plot vs. no treatment

**Fig. 4:**
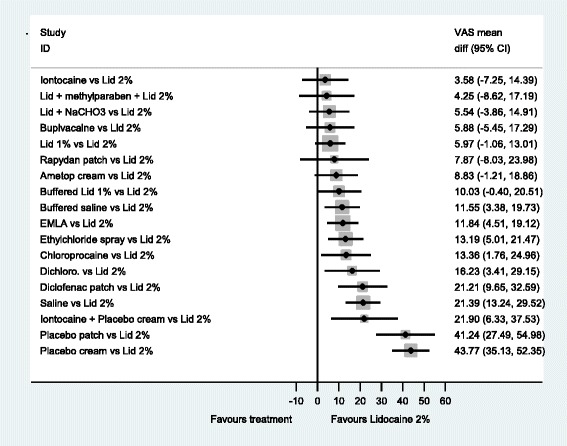
NMA forest plot vs. lidocaine 2 %

**Fig. 5:**
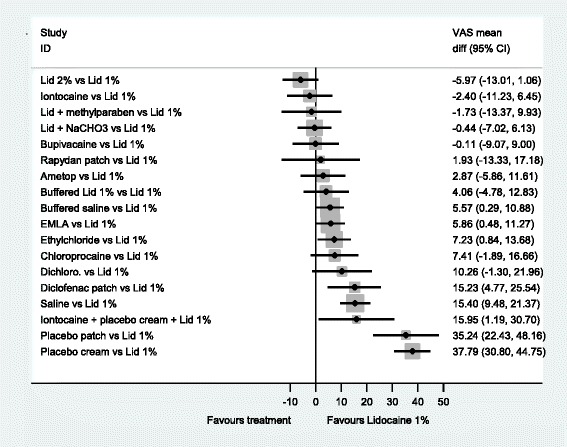
NMA forest plot vs. lidocaine 1 %

**Fig. 6:**
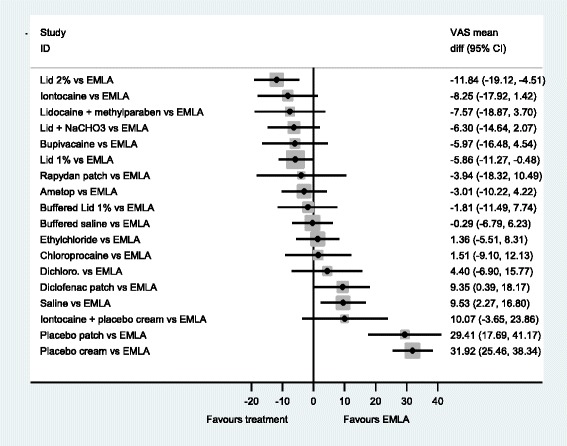
NMA forest plot vs. EMLA cream

**Fig. 7:**
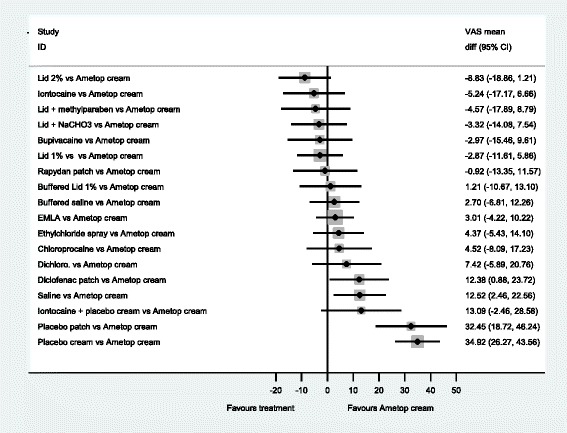
NMA forest plot vs. ametop cream

The forest plots from the NMA results give a clearer picture of the anaesthetics’ relative effectiveness. We examined all comparisons with no treatment, 2 % lidocaine, 1 % lidocaine, EMLA^®^ and Ametop^®^ cream as the comparators, see Figs 3–7. These comparators are chosen because they are commonly used in practice. Forest plots that have the other agents as comparators can be found in Additional file 8.

A word of caution is needed for the interpretation of the forest plots. Frequently the confidence intervals cross zero, casting doubt over the reliability of the point estimate. Even more frequently, the confidence intervals of different comparisons overlap each other; making it difficult to clearly say which is the best treatment. This reflects the finding that no single treatment has a high probability of being the most effective.

When all of the agents are compared with no treatment (Fig. 3), the majority are estimated to be more effective at reducing pain than no treatment. In particular, 2 % lidocaine is estimated as the most effective (mean difference, −25.42 (95 %CI −32.25, −18.57). An examination of this plot shows that members of the ‘caine’ family of drugs are estimated to be much more effective than no treatment, as are Ametop^®^, EMLA^®^ and Rapydan patch.

When 2 % lidocaine is compared with all the other local anaesthetics in a NMA (Fig. 4), the point estimates suggest 2 % lidocaine to be more effective than any other agent. However, the evidence suggests that iontocaine, lid + methyl, Lid + NaCHO3, Bupivacaine, 1 % lid, Rapydan^®^, Ametop^®^ and buffered lidoacine could be as effective as 2 % lidocaine. The evidence suggests that 2 % lidocaine is more effective than diclofenac, saline and placebo treatments.

When all the local anaesthetics are compared to 1 % lidocaine, there is little evidence to suggest that 2 % lidocaine and 1 % lidocaine with additional agents, may be more effective than 1 % lidocaine on its own. However, what is clear is that placebo treatments, diclofenac and saline are likely to be less effective than 1 % lidocaine (Fig. 5).

Local anaesthetic creams are an alternative to injections. Two commonly used are EMLA^®^ and Ametop^®^. Figure 6 has EMLA^®^ as the comparator. This figure shows that EMLA^®^ is likely to be superior to diclofenac, saline, and placebo. Further, this figure indicates that EMLA® is less effective than 2 % lidocaine, but it is unclear whether EMLA^®^ is less effective than Ametop^®^ (−3.01 (−10.22, 4.22)).

When Ametop^®^ cream is the comparator, in Fig. 7 a similar profile is seen to Fig. 7. However, there is a shift in the point estimates that may indicate that overall Ametop^®^ is more effective than EMLA^®^, compared to other agents, at reducing the pain of PVC.

#### Question 2. How does the pain of local anaesthetic application compare with that of routine (non-emergency) unattenuated PVC in adults?

**Fig. 8:**
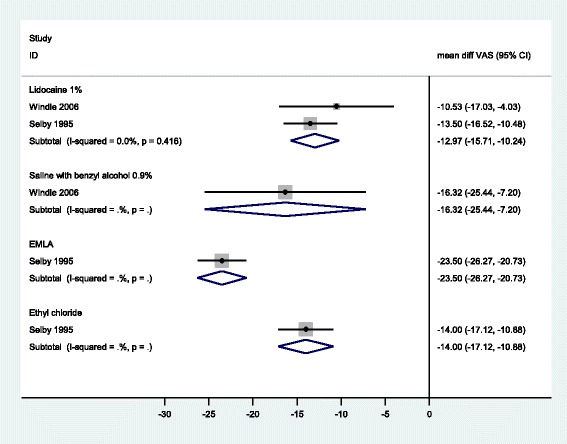
Meta-analysis of studies comparing pain of anaesthetic administration with unattenuated PVC

Six RCTs compared the pain of local anaesthetic administration with that of routine (non-emergency) unattenuated PVC [1, 37, 38, 40, 49, 52]. A summary of these studies results can be found in Additional file 9. However, only two had ‘no treatment’ control groups, which is necessary to address this question [1, 52]. Windle et al. compared 1 % lidocaine and saline with unattenuated PVC; Selby et al. compared 1 % lidocaine, ethyl chloride and EMLA^®^ with unattenuated PVC. For the comparison of 1 % lidocaine with unattenuated PVC, a fixed effects meta-analysis of the data from Windle and Selby was conducted. For all comparisons, the pain of anaesthetic application was less than that of unattenuated PVC. An injection of lidocaine 1 % was estimated to be −12.97 (95 % CI −15.71, −10.24) points as painful, an injection of saline with benzyl alcohol −16.32 (95 % CI −25.44, −7.20) points as painful, ethyl chloride spay −14.00 (95 % CI −17.12, −10.88) points as painful and EMLA^®^ cream −23.50 (95 % CI −26.27, −20.73) points as painful as cannulation without treatment, measured on a 100 mm VAS. See Fig. 8.

Analysis of the consistency of the network indicated some evidence of inconsistency (i.e. direct and indirect evidence suggesting different conclusions), and this was seen for the placebo cream vs no treatment comparison (see Additional file 10).

## Discussion

A total of 37 studies met the inclusion criteria for this review. Twenty seven had data suitable for the NMA and two suitable for the meta-analysis.

The results of the NMA for effectiveness indicate that none of the local anaesthetics can unequivocally be held as the most effective at reducing the pain of PVC. However, the analysis suggests that the majority of active treatments are effective at reducing pain compared to no treatment, with 2 % lidocaine, iontocaine, lid + methyl, Lid + NaCHO3, Bupivacaine, 1 % lid, Rapydan^®^, Ametop^®^ and buffered lidoacine likely to be the most effective.

One unexpected result of the NMA is the apparent greater effectiveness of 1 % lidocaine over 1 % buffered lidocaine (4.16 (95 % CI −4.78, 12.83)). There are a number of possible influences on this result. Firstly, there are no direct comparisons of buffered and normal lidocaine, so this result comes from indirect comparisons. A comparison of estimates for direct and indirect evidence with direct evidence only, show that the same conclusions would be made for either form of evidence for all agents with the exception of placebo cream versus no treatment (Additional file 10). This indicates an increased uncertainty about the NMA results that involve ‘no treatment’ in their network, as is the case with 1 % lidocaine and 1 % buffered lidocaine. This discrepancy may be due to the inability to blind participants who receive no placebo treatment prior to cannulation.

Additionally, the observation from the NMA that Ametop^®^ and EMLA^®^ may be equally effective is an interesting one. This is because Ametop^®^ has both a shorter time of onset (30–45 min compared with 60 min), and provides anaesthesia for longer (4–6 h compared with up to 1 h). Furthermore, Ametop® is a vasodilator, which may aid cannulation, whereas EMLA^®^ is a vasoconstrictor.

Results comparing the pain of local anaesthetic with that of routine (non-emergency) unattenuated PVC in adults (Question 2), suggest that the pain of a local anaesthetic application is considerably less than that of cannulation; this includes injections. This finding counters the objection to providing pain relief for PVC that ‘two sticks are worse than one’. Our evidence suggests that this is not the case and that the ‘caine’ based anaesthetics, should be used to reduce the pain of adult PVC.

These findings are further supported by a survey from Brown (*n* = 180), which examined patient preferences for receiving a lidocaine injection prior to PVC. Participants were assigned to either having or not having lidocaine prior to their PVC. Following cannulation they were asked whether they would like to have this local anaesthetic if they needed PVC again. Brown found that participants who were given lidocaine for their current or previous PVC were more likely to want it next time than those who had never had lidocaine for PVC (current 96 %, previous 80 % and never 50 %). Only 4 % of those who received lidocaine said that they would not want it again [57].

Additional backing for the acceptability of lidocaine injections comes from Levitt et al. who offered a convenience sample of 30 patients the choice of lidocaine, guided imagery or nothing prior to routine PVC. All of those who chose lidocaine said they were satisfied with the intravenous (IV) insertion compared to half of those who chose no pain relief [58].

While this systematic review has the strength of being conducted by an independent research team; it has some limitations. The searches were limited to studies published from 1990 onwards, thus we only included data from the last 25 years. However, 1990 has become a fairly standard cut-off date and it was felt that it was unlikely that any large trials had been missed.

Additionally this systematic review posed a number of challenges to faithfully interpreting the data we found. These included multiple pain models in a variety of participants: different cannula sizes; different anatomical locations; different indications to IV start; no within-participant comparisons of pain intensity; unspecified experience of the cannulator; different ages; genders and unspecified concurrent cognitive behavioural interventions. Further, there were multiple analgesic interventions for the treatment groups and different interventions for the control groups, including: no control intervention, placebo controlled intervention, active control intervention (comparator pharmaceuticals). Four groups received premedication, and there were multiple active interventions with different mechanism of action, and modes of administration (topical/intradermal). In addition, different pain intensity outcome tools were used in the studies that required some to be recalibrated to a metric scale and transforming medians to means. Furthermore, many of the comparisons in the NMA were informed by just one or two studies, with many studies being very small. It is therefore difficult to know whether the lack of evidence for differences between many of the treatments is really a lack of a difference or whether there is not enough power to identify any difference in this analysis. The authors acknowledge that these challenges to synthesizing the data and interpreting the outcomes will increase the uncertainty of our results due to their varying impact on efficacy.

Furthermore, systematic reviews are susceptible to publication bias and bias towards a larger effect size that may come from smaller studies, both of which will favour the intervention. We have not been able to formally investigate the risk of publication bias in this review, and so there may be a possibility that we have overestimated the benefit of local anaesthetic. The searches included those for unpublished studies in an attempt to address this issue; no includable studies were found.

## Conclusions

Routine adult PVC should include local anaesthesia similar to paediatric practice. Evidence suggests that the pain of PVC can be successfully reduced and that the means to do this is either painless or the discomfort of the procedure is acceptable and more acceptable than the pain of PVC. The experience of pain has significance beyond the particular occasion because it can increase anxiety and fear about further treatment and deter people from seeking help in the future [2–5]. The choice of local anaesthetic may be determined by practical and clinical considerations. Hospital protocols and medical and nursing training should be altered to reflect this. The use of local anaesthetic for routine adult PVC should be used as a care quality marker.

### Further research

Resources for further research should be directed towards the following:

- a cost-effectiveness analysis of local anaesthetics including lidocaine injection, Ametop^®^ and EMLA^®^, Including the duration of pain relief and the utility value;
- implementation research to investigate the barriers to changing practice and how these can be overcome; and,
- studies that quantify the impact of local anaesthetic for PVC on long-term needle phobia and seeking medical help for health concerns.

## Additional files

Additional file 1: Searches.docx Search strategy. Literature search strategy. (DOC 207 kb)

Additional file 2: Local anaesthestics used in the studies included in the systematic review.docx Local anaesthetics used in the included studies. Summary local anaesthestics used in the included studies. (DOCX 17 kb)

Additional file 3: Study characteristics.docx Study characteristics Characteristics of included studies. (DOCX 87 kb)

Additional file 4: CONSORT critical appraisal of controlled studies.docx. CONSORT critical appraisal. CONSORT critical appraisal. (DOCX 94 kb)

Additional file 5: STROBE crtiical appraisal of surveys.docx. STROBE critical appraisal. STROBE critical appraisal. (DOCX 32 kb)

Additional file 6: Summary of study results.docx. Summary of study results. Results summary. (DOCX 81 kb)

Additional file 7: Probability of being the most effective.docx Probability of effectiveness. Analysis of probability of most effective drug. (DOCX 13 kb)

Additional file 8: NMA other results plots.docx Results plots from the NMA. Other results plots from the NMA. (DOCX 108 kb)

Additional file 9: Results comparison of the pain of local anaesthetic application with that of peripheral venous cannulation.docx Pain from local anaesthetic vs. pain from peripheral venous cannulation. Comparison of pain from local anaesthestic compared with pain from peripheral venous cannulation. (DOCX 27 kb)

Additional file 10: Direct and indirect evidence only forest plot.docx Direct vs. indirect evidence. Forest plots showing results for indirect and direct evidence. (DOCX 21 kb)

## Abbreviations

ASSIA: Applied Social Sciences Index and Abstracts
CI: Confidence interval
CINAHL: Cumulative Index to Nursing and Allied Health Literature
CONSORT: Consolidated Standards of Reporting Trials
CRD: Centre for Reviews and Dissemination
EMLA: Eutectic mixture of local anaesthetics
HMIC: Health Management Information Consortium
IV: Intravenous
NHS: National Health Service
NICE: National Institute for Health and Care Excellence
NMA: Network meta-analysis
PVC: Peripheral venous cannulation
RCT: Randomised controlled trial
STROBE: STrengthening the Reporting of OBservational studies in Epidemiology
VAS: Visual analogue scale
