# Supplementary material for: First do no harm: pain relief for the peripheral venous cannulation of adults, a systematic review and network meta-analysis
Source: BMC Anesthesiol. 2016 Oct 1;16:81. doi: 10.1186/s12871-016-0252-8 (PMC5045592; doi:10.1186/s12871-016-0252-8)
Supplement: Supplementary file 1 — Searches.docx Search strategy. Literature search strategy. (DOC 207 kb) [file 12871_2016_252_MOESM1_ESM.doc]

**Database Searching**

The initial database searches were conducted in March 2010 using a protocol driven search. The search was conceptualised to use an Intervention and Outcome structure, which took the form of a sensitive list of interventions (interventions, seen as such in this review) – accounting for the English Venous Cannulation and the American Peripheral Catheterization – and outcomes as generated by the question: primarily being adverse events and pain (as in pain caused, pain relief and fear or concern of pain, for example.)

There is the structural concern that, by limiting the search to outcomes, we frame only those outcomes we know to exist at the expense of those we did not know in advance. This concern has been ameliorated as follows:

1) The core MeSH/Emtree controlled syntax term (Catheterization, Peripheral/ (MeSH) vein catheterization/ (Emtree)) were searched as lines in their own right: without any outcome terms. Where outcomes have been used explicitly against the intervention set (after the search of the aforementioned lines) this is to cross-check deficiencies in the indexing of the core controlled syntax terms and focus down the literature base.

2) The outcomes framed by the question were both relatively specific (i.e. Pain) but also relatively sensitive as outcomes. In scoping, it was seen that pain as a concept was relatively well defined in both abstract(s) and title, which ensured the concept of an outcome-based search. The Cochrane library was searched for examples of where Pain (and other outcomes) had been used in reviews (in any context) and we have used controlled syntax and free text examples of 'Pain' to increase sensitivity.

The search syntax was drawn together in draft and circulated to the full project team for comment as well as sign off on the medical/intervention concepts used. 100 records of this draft search were pilot screened by MB and LC to confirm the direction of the search.

The searches were run on March 12^th^ and 13^th^ 2012, June 2013, September 2014 and August 2015. The results were uploaded into Endnote X5 (a variation on the protocol which noted X4: due to a change in the software licence held by the University of Exeter) and de-duplicated before passing to the review team for further de-duplication in RefMAN.

**Web-Searching**

Web-searching was initially conducted on Monday, March 26^th^ and updated in June 2013, September 2014 and August 2015, using a list of defined web-sites and a Google/Meta-search approach. A technical annex is included below detailing the search returns. The following specific web-sites were searched:

- Patients Association
- NICE
- NHS Evidence
- Current Controlled Trials
- Clinical Trials.Gov

Google searching, and a search through the meta-search engine Dogpile, were undertaken with a view to picking up items which were stored outside of these specific web-sites. The Google search was conducted twice; once with a pdf limit on and thereafter through a broader search of the internet (in addition to being an engine used within Dogpile).

The difficulty in the web-searches was defining which sites to search as the topic lacks the generic topical base of diabetes, for example. Venous Cannulation is a process as opposed to a disease area or really, an intervention, in its own right. This explains why the web-searching is not specifically focused to topically relevant web-sites.

| Database | Hits |
| --- | --- |
| 1. [Medline](#medline) | 10111 |
| 1. [Medline in Process](#Process) | 247 |
| 1. [Embase](#Embase) | 8871 |
| 1. [PsycINFO](#PsycINFO) | 287 |
| 1. [HMIC](#HMIC) | 38 |
| 1. [ASSIA](#ASSIA) | 221 |
| 1. [British Nursing Index (BNI)](#BNI) | 298 (searched via NHS Evidence) |
| 1. [Web of Science](#wos) | 4922 |
| 1. [Sociological Abstracts](#Sociological) | 2 |
| 1. [Social Policy and Practice](#Practice) | 3 |
| 1. [Cochrane Library (ALL)](#Cochrane) | 1169 (Reviews: 20 DARE: 13 Central: 1103 Methods: 6 HTA: 9 NHS EEDS: 18) |
| 1. [CINAHL](#Cinahl) | 179 (934) |
| Total | 26348 |
| - Endnote De-duplication | - 6952 |
| - RefMan De-duplication | - 3,028 (2,649 (auto de-dup) + 379 (manual de-dup)) |
| Unique References to Screen | 16,368 |

**1.**

**Database:** Medline

**Host:** OVID

**Data Parameters:** 1946 to February Week 5 2012

**Date Searched:** Monday, March 12^th^ 2012

**Searcher:** Cooper

**Hits:** 10111

**Strategy:**

1. [Catheterization, Peripheral](http://ovidsp.uk.ovid.com/sp-3.5.1a/ovidweb.cgi?&S=KPDDPDOEHHHFENHAFNALOCAGNFOGAA00&Search+Link=*"Catheterization%2C+Peripheral"%2F)/
2. [Catheterization, Peripheral](http://ovidsp.uk.ovid.com/sp-3.5.1a/ovidweb.cgi?&S=KPDDPDOEHHHFENHAFNALOCAGNFOGAA00&Search+Link=*"Catheterization%2C+Peripheral"%2F)/
3. ((venous adj3 Catheter$) and peripheral).ti,ab.
4. PIVC.ti,ab.
5. (venous adj3 Cannula$).ti,ab.
6. ((vein$ adj3 (Catheter$)) and (peripheral)).ti,ab.
7. (vein$ adj3 cannula$).ti,ab.
8. ((intravenous or IV) adj3 cannula$).ti,ab.
9. ((intravenous or IV) adj3 catheter$).ti,ab.
10. ((multiple or repeat$) adj3 (cannula$ or Catheter$)).ti,ab.
11. ((cannula$ or cather$) adj3 (puncture$ or insert$ or needle)).ti,ab.
12. (Venipuncture or venepuncture).ti,ab.
13. [*Punctures](http://ovidsp.uk.ovid.com/sp-3.5.1a/ovidweb.cgi?&S=PHBDPDKCLMHFIMKEFNALIAEGMOHMAA00&Search+Link=*"Punctures"%2F)/
14. Or/2-13
15. ae.fs.
16. (adverse adj3 event$).ti,ab.
17. (complication$ or infection$ or prepar$ or compliance).ti,ab.
18. (infiltration or extravasation).ti,ab.
19. Thrombophlebitis/
20. (phlebitis or thrombophlebitis or (VIP)).ti,ab.
21. *teaching/
22. *education/
23. (train$ or teach$ or taught or learn$).ti,ab.
24. exp Pain/
25. Pain Management/
26. pain$.mp.
27. (discomfort or irritation or success$ or attempt$ or error$ or mistake$ or refuse or refusal).ti,ab.
28. risk/
29. exp Emotions/
30. (anxiety or anxious$ or concern$ or worry or worried or fear$ or phobia$ or distress$ or coping or trauma$ or stress$ or suffer$ or experience).ti,ab.
31. exp "Patient Acceptance of Health Care"/
32. exp patient satisfaction/
33. (patient adj3 (preference or satisf$)).ti,ab.
34. (quality adj3 life).ti,ab.
35. Or/15-34
36. 14 and 35
37. 1 or 36
38. limit 18 to yr="1990 -Current"

**Notes:** Search limited by date and to human only populations.

**File Name:** Medline

**2.**

**Database:** Medline in Process

**Host:** OVID

**Data Parameters:** March 09, 2012

**Date Searched:** Monday, March 12^th^ 2012

**Searcher:** Cooper

**Hits:** 247

**Strategy:**

1. [Catheterization, Peripheral](http://ovidsp.uk.ovid.com/sp-3.5.1a/ovidweb.cgi?&S=KPDDPDOEHHHFENHAFNALOCAGNFOGAA00&Search+Link=*"Catheterization%2C+Peripheral"%2F)/
2. [Catheterization, Peripheral](http://ovidsp.uk.ovid.com/sp-3.5.1a/ovidweb.cgi?&S=KPDDPDOEHHHFENHAFNALOCAGNFOGAA00&Search+Link=*"Catheterization%2C+Peripheral"%2F)/
3. ((venous adj3 Catheter$) and peripheral).ti,ab.
4. PIVC.ti,ab.
5. (venous adj3 Cannula$).ti,ab.
6. ((vein$ adj3 (Catheter$)) and (peripheral)).ti,ab.
7. (vein$ adj3 cannula$).ti,ab.
8. ((intravenous or IV) adj3 cannula$).ti,ab.
9. ((intravenous or IV) adj3 catheter$).ti,ab.
10. ((multiple or repeat$) adj3 (cannula$ or Catheter$)).ti,ab.
11. ((cannula$ or cather$) adj3 (puncture$ or insert$ or needle)).ti,ab.
12. (Venipuncture or venepuncture).ti,ab.
13. [*Punctures](http://ovidsp.uk.ovid.com/sp-3.5.1a/ovidweb.cgi?&S=PHBDPDKCLMHFIMKEFNALIAEGMOHMAA00&Search+Link=*"Punctures"%2F)/
14. Or/2-13
15. ae.fs.
16. (adverse adj3 event$).ti,ab.
17. (complication$ or infection$ or prepar$ or compliance).ti,ab.
18. (infiltration or extravasation).ti,ab.
19. Thrombophlebitis/
20. (phlebitis or thrombophlebitis or (VIP)).ti,ab.
21. *teaching/
22. *education/
23. (train$ or teach$ or taught or learn$).ti,ab.
24. exp Pain/
25. Pain Management/
26. pain$.mp.
27. (discomfort or irritation or success$ or attempt$ or error$ or mistake$ or refuse or refusal).ti,ab.
28. risk/
29. exp Emotions/
30. (anxiety or anxious$ or concern$ or worry or worried or fear$ or phobia$ or distress$ or coping or trauma$ or stress$ or suffer$ or experience).ti,ab.
31. exp "Patient Acceptance of Health Care"/
32. exp patient satisfaction/
33. (patient adj3 (preference or satisf$)).ti,ab.
34. (quality adj3 life).ti,ab.
35. Or/15-34
36. 14 and 35
37. 1 or 36
38. limit 18 to yr="1990 -Current"

**Notes:** Search limited by date and to human only populations.

**File Name:** Medline in Process

**3.**

**Database:** Embase

**Host:** OVID

**Data Parameters:** 1980 to 2012 Week 10

**Date Searched:** Tuesday, March 13^th^ 2012

**Searcher:** Cooper

**Hits:**

**Strategy:**

1. vein catheterization/
2. catheterization/ and (vein$ or venous or peripheral).ti,ab.
3. [cannulation](http://ovidsp.uk.ovid.com/sp-3.5.1a/ovidweb.cgi?&S=OMOFPDOOMJHFAKDJFNALFHEGAJHOAA00&Search+Link="cannulation"%2F)/ and (vein$ or venous or peripheral).ti,ab.
4. ((venous adj3 Catheter$) and peripheral).ti,ab.
5. PIVC.ti,ab.
6. (venous adj3 Cannula$).ti,ab.
7. (vein$ adj3 cannula$).ti,ab.
8. ((vein$ adj3 (Catheter$)) and (peripheral)).ti,ab.
9. ((intravenous or IV) adj3 cannula$).ti,ab.
10. intravenous catheter/
11. ((intravenous or IV) adj3 catheter$).ti,ab.
12. ((multiple or repeat$) adj3 (cannula$ or Catheter$)).ti,ab.
13. ((cannula$ or cather$) adj3 (puncture$ or insert$ or needle)).ti,ab.
14. (Venipuncture or venepuncture).ti,ab.
15. Or/1-14
16. ae.fs.
17. (adverse adj3 event$).ti,ab.
18. (complication$ or infection$ or prepar$ or compliance).ti,ab.
19. (infiltration or extravasation).ti,ab.
20. Thrombophlebitis/
21. (phlebitis or thrombophlebitis or (VIP)).ti,ab.
22. exp education/
23. (train$ or teach$ or taught or learn$).ti,ab.
24. pain/
25. pain$.mp.
26. (discomfort or irritation or success$ or attempt$ or error$ or mistake$ or refuse or refusal).ti,ab.
27. exp emotion/
28. (anxiety or anxious$ or concern$ or worry or worried or fear$ or phobia$ or distress$ or coping or trauma$ or stress$ or suffer$ or experience).ti,ab.
29. exp patient attitude/
30. (patient adj3 (preference or satisf$)).ti,ab.
31. (quality adj3 life).ti,ab.
32. Or/16-31

**Notes:** Search limited by date and to human only populations.

**File Name:** Embase

**4.**

**Database:** PsycINFO

**Host:** OVID

**Data Parameters:** 1806 to March Week 1 2012

**Date Searched:** Monday, March 12^th^ 2012

**Searcher:** Cooper

**Hits:** 287

**Strategy:**

1. [Catheterization, Peripheral](http://ovidsp.uk.ovid.com/sp-3.5.1a/ovidweb.cgi?&S=KPDDPDOEHHHFENHAFNALOCAGNFOGAA00&Search+Link=*"Catheterization%2C+Peripheral"%2F)/
2. [Catheterization, Peripheral](http://ovidsp.uk.ovid.com/sp-3.5.1a/ovidweb.cgi?&S=KPDDPDOEHHHFENHAFNALOCAGNFOGAA00&Search+Link=*"Catheterization%2C+Peripheral"%2F)/
3. ((venous adj3 Catheter$) and peripheral).ti,ab.
4. PIVC.ti,ab.
5. (venous adj3 Cannula$).ti,ab.
6. ((vein$ adj3 (Catheter$)) and (peripheral)).ti,ab.
7. (vein$ adj3 cannula$).ti,ab.
8. ((intravenous or IV) adj3 cannula$).ti,ab.
9. ((intravenous or IV) adj3 catheter$).ti,ab.
10. ((multiple or repeat$) adj3 (cannula$ or Catheter$)).ti,ab.
11. ((cannula$ or cather$) adj3 (puncture$ or insert$ or needle)).ti,ab.
12. (Venipuncture or venepuncture).ti,ab.
13. [*Punctures](http://ovidsp.uk.ovid.com/sp-3.5.1a/ovidweb.cgi?&S=PHBDPDKCLMHFIMKEFNALIAEGMOHMAA00&Search+Link=*"Punctures"%2F)/
14. Or/2-13
15. ae.fs.
16. (adverse adj3 event$).ti,ab.
17. (complication$ or infection$ or prepar$ or compliance).ti,ab.
18. (infiltration or extravasation).ti,ab.
19. Thrombophlebitis/
20. (phlebitis or thrombophlebitis or (VIP)).ti,ab.
21. *teaching/
22. *education/
23. (train$ or teach$ or taught or learn$).ti,ab.
24. exp Pain/
25. Pain Management/
26. pain$.mp.
27. (discomfort or irritation or success$ or attempt$ or error$ or mistake$ or refuse or refusal).ti,ab.
28. risk/
29. exp Emotions/
30. (anxiety or anxious$ or concern$ or worry or worried or fear$ or phobia$ or distress$ or coping or trauma$ or stress$ or suffer$ or experience).ti,ab.
31. exp "Patient Acceptance of Health Care"/
32. exp patient satisfaction/
33. (patient adj3 (preference or satisf$)).ti,ab.
34. (quality adj3 life).ti,ab.
35. Or/15-34
36. 14 and 35
37. 1 or 36
38. limit 18 to yr="1990 -Current"

**Notes:** Search limited by date and to human only populations.

**File Name:** PsycINFO

**5.**

**Database:** HMIC

**Host:** OVID

**Data Parameters:** 1979 to January 2012

**Date Searched:** Monday, March 12^th^ 2012

**Searcher:** Cooper

**Hits:** 38

**Strategy:**

1. [Catheterization, Peripheral](http://ovidsp.uk.ovid.com/sp-3.5.1a/ovidweb.cgi?&S=KPDDPDOEHHHFENHAFNALOCAGNFOGAA00&Search+Link=*"Catheterization%2C+Peripheral"%2F)/
2. [Catheterization, Peripheral](http://ovidsp.uk.ovid.com/sp-3.5.1a/ovidweb.cgi?&S=KPDDPDOEHHHFENHAFNALOCAGNFOGAA00&Search+Link=*"Catheterization%2C+Peripheral"%2F)/
3. ((venous adj3 Catheter$) and peripheral).ti,ab.
4. PIVC.ti,ab.
5. (venous adj3 Cannula$).ti,ab.
6. ((vein$ adj3 (Catheter$)) and (peripheral)).ti,ab.
7. (vein$ adj3 cannula$).ti,ab.
8. ((intravenous or IV) adj3 cannula$).ti,ab.
9. ((intravenous or IV) adj3 catheter$).ti,ab.
10. ((multiple or repeat$) adj3 (cannula$ or Catheter$)).ti,ab.
11. ((cannula$ or cather$) adj3 (puncture$ or insert$ or needle)).ti,ab.
12. (Venipuncture or venepuncture).ti,ab.
13. [*Punctures](http://ovidsp.uk.ovid.com/sp-3.5.1a/ovidweb.cgi?&S=PHBDPDKCLMHFIMKEFNALIAEGMOHMAA00&Search+Link=*"Punctures"%2F)/
14. Or/2-13
15. ae.fs.
16. (adverse adj3 event$).ti,ab.
17. (complication$ or infection$ or prepar$ or compliance).ti,ab.
18. (infiltration or extravasation).ti,ab.
19. Thrombophlebitis/
20. (phlebitis or thrombophlebitis or (VIP)).ti,ab.
21. *teaching/
22. *education/
23. (train$ or teach$ or taught or learn$).ti,ab.
24. exp Pain/
25. Pain Management/
26. pain$.mp.
27. (discomfort or irritation or success$ or attempt$ or error$ or mistake$ or refuse or refusal).ti,ab.
28. risk/
29. exp Emotions/
30. (anxiety or anxious$ or concern$ or worry or worried or fear$ or phobia$ or distress$ or coping or trauma$ or stress$ or suffer$ or experience).ti,ab.
31. exp "Patient Acceptance of Health Care"/
32. exp patient satisfaction/
33. (patient adj3 (preference or satisf$)).ti,ab.
34. (quality adj3 life).ti,ab.
35. Or/15-34
36. 14 and 35
37. 1 or 36
38. limit 18 to yr="1990 -Current"

**Notes:** Search limited by date and to human only populations.

**File Name:** HMIC

**6.**

**Database:** ASSIA

**Host:** CSA

**Data Parameters:** 1979 to January 2012

**Date Searched:** Monday, March 12^th^ 2012

**Searcher:** Cooper

**Hits:** 221

**Strategy:**

1. DE="**cannulas**"
2. DE="catheterization"
3. ((KW=(**venous** within 3 **Catheter***)) and KW=(**peripheral**))
4. ((KW=(**vein*** within 3 **Catheter***)) and KW=(**peripheral**))
5. ((multiple or repeat*) within 3 (Catheter*))
6. Cannula*
7. ((intravenous or IV) within 3 cannula*)
8. ((intravenous or IV) within 3 catheter*)
9. ((cannula* or cather*) within 3 (puncture* or insert* or needle))
10. (Venipuncture or venepuncture)
11. Or/
12. (adverse within 3 event*)
13. (complication* or infection* or prepar* or compliance or infiltration or extravasation or phlebitis or thrombophlebitis or (VIP) or train* or teach* or taught or learn* or pain* or discomfort or irritation or success* or attempt* or error* or mistake* or refuse or refusal or anxiety or anxious* or concern* or worry or worried or fear* or phobia* or distress* or coping or trauma* or stress* or suffer* or experience)
14. (patient within 3 (preference or satisf*))
15. (quality within 3 life)
16. Or/2-5

**Notes:** Search limited by date.

**File Name:** Assia

**7.**

**Database:** British Nursing Index

**Host:** NHS Evidence

**Data Parameters:**

**Date Searched:** Monday, March 12^th^ 2012

**Searcher:** Cooper

**Hits:** 298

**Strategy:**

1. (((venous adj3 Catheter*) AND peripheral)).ti,ab
2. PIVC.ti,ab
3. ((venous adj3 Cannula*)).ti,ab
4. (((vein* adj3 (Catheter*)) AND (peripheral))).ti,ab
5. ((vein* adj3 cannula*)).ti,ab
6. (((intravenous OR IV) adj3 cannula*)).ti,ab
7. (((intravenous OR IV) adj3 catheter*)).ti,ab
8. (((multiple OR repeat*) adj3 (cannula* OR Catheter*))).ti,ab
9. cannula* OR cather*) adj3 (puncture* OR insert* OR needle))).ti,ab
10. ((Venipuncture OR venepuncture)).ti,ab
11. Or/1-10

**Notes:** N/A

**File Name:** BNI

**8.**

**Database:** Web of Science (SCI-EXPANDED, SSCI, A&HCI, CPCI-S, CPCI-SSH.)

**Host:** ISI

**Data Parameters:** 1899-Current

**Date Searched:** Monday, March 12^th^ 2012

**Searcher:** Cooper

**Hits:** 4922

**Strategy:**

1. (((venous Near/3 Catheter*) AND peripheral))
2. PIVC
3. ((venous Near/3 Cannula*))
4. (((vein* Near/3 (Catheter*)) AND (peripheral)))
5. ((vein* Near/3 cannula*))
6. (((intravenous OR IV) Near/3 cannula*))
7. (((intravenous OR IV) Near/3 catheter*))
8. (((multiple OR repeat*) Near/3 (cannula* OR Catheter*)))
9. ((cannula* OR cather*) Near/3 (puncture* OR insert* OR needle))
10. ((Venipuncture OR venepuncture))
11. Or/1-10
12. (adverse Near/3 event*)
13. (complication* or infection* or prepar* or compliance or infiltration or extravasation or phlebitis or thrombophlebitis or (VIP) or train* or teach* or taught or learn* or pain* or discomfort or irritation or success* or attempt* or error* or mistake* or refuse or refusal or anxiety or anxious* or concern* or worry or worried or fear* or phobia* or distress* or coping or trauma* or stress* or suffer* or experience)
14. (patient Near/3 (preference or satisf*))
15. (quality Near/3 life)
16. Or/12-15
17. 11 and 16
18. Limit to Humans
19. Limit to 1990-2012

**Notes:** Search limited by date and to human only populations.

**File Name:** WOS

**9.**

**Database:** Sociological Abstracts

**Host:** CSA

**Data Parameters:**

**Date Searched:** Monday, March 12^th^ 2012

**Searcher:** Cooper

**Hits:** 2

**Strategy:**

1. DE="**cannulas**"
2. DE="catheterization"
3. ((KW=(**venous** within 3 **Catheter***)) and KW=(**peripheral**))
4. ((KW=(**vein*** within 3 **Catheter***)) and KW=(**peripheral**))
5. ((multiple or repeat*) within 3 (Catheter*))
6. Cannula*
7. ((intravenous or IV) within 3 cannula*)
8. ((intravenous or IV) within 3 catheter*)
9. ((cannula* or cather*) within 3 (puncture* or insert* or needle))
10. (Venipuncture or venepuncture)
11. Or/
12. (adverse within 3 event*)
13. (complication* or infection* or prepar* or compliance or infiltration or extravasation or phlebitis or thrombophlebitis or (VIP) or train* or teach* or taught or learn* or pain* or discomfort or irritation or success* or attempt* or error* or mistake* or refuse or refusal or anxiety or anxious* or concern* or worry or worried or fear* or phobia* or distress* or coping or trauma* or stress* or suffer* or experience)
14. (patient within 3 (preference or satisf*))
15. (quality within 3 life)
16. Or/2-5

**Notes:** N/A

**File Name:** Soc Abs

**10.**

**Database:** Social Policy and Practice

**Host:** OVID

**Data Parameters:** 201201

**Date Searched:** Monday, March 12^th^ 2012

**Searcher:** Cooper

**Hits:** 3

**Strategy:**

1. [Catheterization, Peripheral](http://ovidsp.uk.ovid.com/sp-3.5.1a/ovidweb.cgi?&S=KPDDPDOEHHHFENHAFNALOCAGNFOGAA00&Search+Link=*"Catheterization%2C+Peripheral"%2F)/
2. [Catheterization, Peripheral](http://ovidsp.uk.ovid.com/sp-3.5.1a/ovidweb.cgi?&S=KPDDPDOEHHHFENHAFNALOCAGNFOGAA00&Search+Link=*"Catheterization%2C+Peripheral"%2F)/
3. ((venous adj3 Catheter$) and peripheral).ti,ab.
4. PIVC.ti,ab.
5. (venous adj3 Cannula$).ti,ab.
6. ((vein$ adj3 (Catheter$)) and (peripheral)).ti,ab.
7. (vein$ adj3 cannula$).ti,ab.
8. ((intravenous or IV) adj3 cannula$).ti,ab.
9. ((intravenous or IV) adj3 catheter$).ti,ab.
10. ((multiple or repeat$) adj3 (cannula$ or Catheter$)).ti,ab.
11. ((cannula$ or cather$) adj3 (puncture$ or insert$ or needle)).ti,ab.
12. (Venipuncture or venepuncture).ti,ab.
13. [*Punctures](http://ovidsp.uk.ovid.com/sp-3.5.1a/ovidweb.cgi?&S=PHBDPDKCLMHFIMKEFNALIAEGMOHMAA00&Search+Link=*"Punctures"%2F)/
14. Or/2-13
15. ae.fs.
16. (adverse adj3 event$).ti,ab.
17. (complication$ or infection$ or prepar$ or compliance).ti,ab.
18. (infiltration or extravasation).ti,ab.
19. Thrombophlebitis/
20. (phlebitis or thrombophlebitis or (VIP)).ti,ab.
21. *teaching/
22. *education/
23. (train$ or teach$ or taught or learn$).ti,ab.
24. exp Pain/
25. Pain Management/
26. pain$.mp.
27. (discomfort or irritation or success$ or attempt$ or error$ or mistake$ or refuse or refusal).ti,ab.
28. risk/
29. exp Emotions/
30. (anxiety or anxious$ or concern$ or worry or worried or fear$ or phobia$ or distress$ or coping or trauma$ or stress$ or suffer$ or experience).ti,ab.
31. exp "Patient Acceptance of Health Care"/
32. exp patient satisfaction/
33. (patient adj3 (preference or satisf$)).ti,ab.
34. (quality adj3 life).ti,ab.
35. Or/15-34
36. 14 and 35
37. 1 or 36
38. limit 18 to yr="1990 -Current"

**Notes:** Search limited by date and to human only populations.

**File Name:** Medline in Process

**11.**

**Database:** Cochrane Library (ALL)

**Host:** <http://www.thecochranelibrary.com/view/0/index.html>

**Data Parameters: Issue 2 of 12, Feb 2012**

**Date Searched:** Monday, March 12^th^ 2012

**Searcher:** Cooper

**Hits**: 1169

**Strategy:**

| **ID** | **Search** | **Hits** | **Edit** | **Delete** |
| --- | --- | --- | --- | --- |
| #1 | [MeSH descriptor **Catheterization, Peripheral**, this term only](http://onlinelibrary.wiley.com/o/cochrane/searchHistory?mode=runquery&qnum=1) | 498 | [edit](http://onlinelibrary.wiley.com/search-web/cochrane/searchHistory?mode=editquery&qnum=1&searchKey=bdf8bd7a-a5c3-4b37-8fd0-201eeb31d073) | [delete](http://onlinelibrary.wiley.com/search-web/cochrane/searchHistory?mode=deletequery&qnum=1&uuid=bdf8bd7a-a5c3-4b37-8fd0-201eeb31d073&searchKey=bdf8bd7a-a5c3-4b37-8fd0-201eeb31d073) |
| #2 | [MeSH descriptor **Catheterization, Peripheral**, this term only](http://onlinelibrary.wiley.com/o/cochrane/searchHistory?mode=runquery&qnum=2) | 498 | [edit](http://onlinelibrary.wiley.com/search-web/cochrane/searchHistory?mode=editquery&qnum=2&searchKey=bdf8bd7a-a5c3-4b37-8fd0-201eeb31d073) | [delete](http://onlinelibrary.wiley.com/search-web/cochrane/searchHistory?mode=deletequery&qnum=2&uuid=bdf8bd7a-a5c3-4b37-8fd0-201eeb31d073&searchKey=bdf8bd7a-a5c3-4b37-8fd0-201eeb31d073) |
| #3 | [((venous Near/3 Catheter*) and peripheral):ti,ab,kw](http://onlinelibrary.wiley.com/o/cochrane/searchHistory?mode=runquery&qnum=3) | 141 | [edit](javascript:doPopup('/search-web/cochrane/searchHistory?mode=editquery&qnum=3&searchKey=bdf8bd7a-a5c3-4b37-8fd0-201eeb31d073', 400)) | [delete](http://onlinelibrary.wiley.com/search-web/cochrane/searchHistory?mode=deletequery&qnum=3&uuid=bdf8bd7a-a5c3-4b37-8fd0-201eeb31d073&searchKey=bdf8bd7a-a5c3-4b37-8fd0-201eeb31d073) |
| #4 | [(PIVC):ti,ab,kw](http://onlinelibrary.wiley.com/o/cochrane/searchHistory?mode=runquery&qnum=4) | 1 | [edit](javascript:doPopup('/search-web/cochrane/searchHistory?mode=editquery&qnum=4&searchKey=bdf8bd7a-a5c3-4b37-8fd0-201eeb31d073', 400)) | [delete](http://onlinelibrary.wiley.com/search-web/cochrane/searchHistory?mode=deletequery&qnum=4&uuid=bdf8bd7a-a5c3-4b37-8fd0-201eeb31d073&searchKey=bdf8bd7a-a5c3-4b37-8fd0-201eeb31d073) |
| #5 | [(venous Near/3 Cannula*):ti,ab,kw](http://onlinelibrary.wiley.com/o/cochrane/searchHistory?mode=runquery&qnum=5) | 184 | [edit](javascript:doPopup('/search-web/cochrane/searchHistory?mode=editquery&qnum=5&searchKey=bdf8bd7a-a5c3-4b37-8fd0-201eeb31d073', 400)) | [delete](http://onlinelibrary.wiley.com/search-web/cochrane/searchHistory?mode=deletequery&qnum=5&uuid=bdf8bd7a-a5c3-4b37-8fd0-201eeb31d073&searchKey=bdf8bd7a-a5c3-4b37-8fd0-201eeb31d073) |
| #6 | [((vein* Near/3 (Catheter*)) and (peripheral)):ti,ab,kw](http://onlinelibrary.wiley.com/o/cochrane/searchHistory?mode=runquery&qnum=6) | 26 | [edit](javascript:doPopup('/search-web/cochrane/searchHistory?mode=editquery&qnum=6&searchKey=bdf8bd7a-a5c3-4b37-8fd0-201eeb31d073', 400)) | [delete](http://onlinelibrary.wiley.com/search-web/cochrane/searchHistory?mode=deletequery&qnum=6&uuid=bdf8bd7a-a5c3-4b37-8fd0-201eeb31d073&searchKey=bdf8bd7a-a5c3-4b37-8fd0-201eeb31d073) |
| #7 | [(vein* Near/3 cannula*) :ti,ab,kw](http://onlinelibrary.wiley.com/o/cochrane/searchHistory?mode=runquery&qnum=7) | 79 | [edit](javascript:doPopup('/search-web/cochrane/searchHistory?mode=editquery&qnum=7&searchKey=bdf8bd7a-a5c3-4b37-8fd0-201eeb31d073', 400)) | [delete](http://onlinelibrary.wiley.com/search-web/cochrane/searchHistory?mode=deletequery&qnum=7&uuid=bdf8bd7a-a5c3-4b37-8fd0-201eeb31d073&searchKey=bdf8bd7a-a5c3-4b37-8fd0-201eeb31d073) |
| #8 | [((intravenous or IV) Near/3 cannula*) :ti,ab,kw](http://onlinelibrary.wiley.com/o/cochrane/searchHistory?mode=runquery&qnum=8) | 181 | [edit](javascript:doPopup('/search-web/cochrane/searchHistory?mode=editquery&qnum=8&searchKey=bdf8bd7a-a5c3-4b37-8fd0-201eeb31d073', 400)) | [delete](http://onlinelibrary.wiley.com/search-web/cochrane/searchHistory?mode=deletequery&qnum=8&uuid=bdf8bd7a-a5c3-4b37-8fd0-201eeb31d073&searchKey=bdf8bd7a-a5c3-4b37-8fd0-201eeb31d073) |
| #9 | [((intravenous or IV) Near/3 catheter*):ti,ab,kw](http://onlinelibrary.wiley.com/o/cochrane/searchHistory?mode=runquery&qnum=9) | 306 | [edit](javascript:doPopup('/search-web/cochrane/searchHistory?mode=editquery&qnum=9&searchKey=bdf8bd7a-a5c3-4b37-8fd0-201eeb31d073', 400)) | [delete](http://onlinelibrary.wiley.com/search-web/cochrane/searchHistory?mode=deletequery&qnum=9&uuid=bdf8bd7a-a5c3-4b37-8fd0-201eeb31d073&searchKey=bdf8bd7a-a5c3-4b37-8fd0-201eeb31d073) |
| #10 | [((multiple or repeat*) Near/3 (cannula* or Catheter*)):ti,ab,kw](http://onlinelibrary.wiley.com/o/cochrane/searchHistory?mode=runquery&qnum=10) | 86 | [edit](javascript:doPopup('/search-web/cochrane/searchHistory?mode=editquery&qnum=10&searchKey=bdf8bd7a-a5c3-4b37-8fd0-201eeb31d073', 400)) | [delete](http://onlinelibrary.wiley.com/search-web/cochrane/searchHistory?mode=deletequery&qnum=10&uuid=bdf8bd7a-a5c3-4b37-8fd0-201eeb31d073&searchKey=bdf8bd7a-a5c3-4b37-8fd0-201eeb31d073) |
| #11 | [((cannula* or cather*) Near/3 (puncture* or insert* or needle)):ti,ab,kw](http://onlinelibrary.wiley.com/o/cochrane/searchHistory?mode=runquery&qnum=11) | 151 | [edit](javascript:doPopup('/search-web/cochrane/searchHistory?mode=editquery&qnum=11&searchKey=bdf8bd7a-a5c3-4b37-8fd0-201eeb31d073', 400)) | [delete](http://onlinelibrary.wiley.com/search-web/cochrane/searchHistory?mode=deletequery&qnum=11&uuid=bdf8bd7a-a5c3-4b37-8fd0-201eeb31d073&searchKey=bdf8bd7a-a5c3-4b37-8fd0-201eeb31d073) |
| #12 | [(Venipuncture or venepuncture) :ti,ab,kw](http://onlinelibrary.wiley.com/o/cochrane/searchHistory?mode=runquery&qnum=12) | 387 | [edit](javascript:doPopup('/search-web/cochrane/searchHistory?mode=editquery&qnum=12&searchKey=bdf8bd7a-a5c3-4b37-8fd0-201eeb31d073', 400)) | [delete](http://onlinelibrary.wiley.com/search-web/cochrane/searchHistory?mode=deletequery&qnum=12&uuid=bdf8bd7a-a5c3-4b37-8fd0-201eeb31d073&searchKey=bdf8bd7a-a5c3-4b37-8fd0-201eeb31d073) |
| #13 | [(adverse Near/3 event*):ti,ab,kw](http://onlinelibrary.wiley.com/o/cochrane/searchHistory?mode=runquery&qnum=13) | 24580 | [edit](javascript:doPopup('/search-web/cochrane/searchHistory?mode=editquery&qnum=13&searchKey=bdf8bd7a-a5c3-4b37-8fd0-201eeb31d073', 400)) | [delete](http://onlinelibrary.wiley.com/search-web/cochrane/searchHistory?mode=deletequery&qnum=13&uuid=bdf8bd7a-a5c3-4b37-8fd0-201eeb31d073&searchKey=bdf8bd7a-a5c3-4b37-8fd0-201eeb31d073) |
| #14 | [(complication* or infection* or prepar* or compliance):ti,ab,kw](http://onlinelibrary.wiley.com/o/cochrane/searchHistory?mode=runquery&qnum=14) | 137672 | [edit](javascript:doPopup('/search-web/cochrane/searchHistory?mode=editquery&qnum=14&searchKey=bdf8bd7a-a5c3-4b37-8fd0-201eeb31d073', 400)) | [delete](http://onlinelibrary.wiley.com/search-web/cochrane/searchHistory?mode=deletequery&qnum=14&uuid=bdf8bd7a-a5c3-4b37-8fd0-201eeb31d073&searchKey=bdf8bd7a-a5c3-4b37-8fd0-201eeb31d073) |
| #15 | [(infiltration or extravasation) :ti,ab,kw](http://onlinelibrary.wiley.com/o/cochrane/searchHistory?mode=runquery&qnum=15) | 2231 | [edit](javascript:doPopup('/search-web/cochrane/searchHistory?mode=editquery&qnum=15&searchKey=bdf8bd7a-a5c3-4b37-8fd0-201eeb31d073', 400)) | [delete](http://onlinelibrary.wiley.com/search-web/cochrane/searchHistory?mode=deletequery&qnum=15&uuid=bdf8bd7a-a5c3-4b37-8fd0-201eeb31d073&searchKey=bdf8bd7a-a5c3-4b37-8fd0-201eeb31d073) |
| #16 | [MeSH descriptor **Thrombophlebitis**, this term only](http://onlinelibrary.wiley.com/o/cochrane/searchHistory?mode=runquery&qnum=16) | 1089 | [edit](http://onlinelibrary.wiley.com/search-web/cochrane/searchHistory?mode=editquery&qnum=16&searchKey=bdf8bd7a-a5c3-4b37-8fd0-201eeb31d073) | [delete](http://onlinelibrary.wiley.com/search-web/cochrane/searchHistory?mode=deletequery&qnum=16&uuid=bdf8bd7a-a5c3-4b37-8fd0-201eeb31d073&searchKey=bdf8bd7a-a5c3-4b37-8fd0-201eeb31d073) |
| #17 | [(phlebitis or thrombophlebitis or (VIP)):ti,ab,kw](http://onlinelibrary.wiley.com/o/cochrane/searchHistory?mode=runquery&qnum=17) | 1941 | [edit](javascript:doPopup('/search-web/cochrane/searchHistory?mode=editquery&qnum=17&searchKey=bdf8bd7a-a5c3-4b37-8fd0-201eeb31d073', 400)) | [delete](http://onlinelibrary.wiley.com/search-web/cochrane/searchHistory?mode=deletequery&qnum=17&uuid=bdf8bd7a-a5c3-4b37-8fd0-201eeb31d073&searchKey=bdf8bd7a-a5c3-4b37-8fd0-201eeb31d073) |
| #18 | [MeSH descriptor **Teaching**, this term only](http://onlinelibrary.wiley.com/o/cochrane/searchHistory?mode=runquery&qnum=18) | 1130 | [edit](http://onlinelibrary.wiley.com/search-web/cochrane/searchHistory?mode=editquery&qnum=18&searchKey=bdf8bd7a-a5c3-4b37-8fd0-201eeb31d073) | [delete](http://onlinelibrary.wiley.com/search-web/cochrane/searchHistory?mode=deletequery&qnum=18&uuid=bdf8bd7a-a5c3-4b37-8fd0-201eeb31d073&searchKey=bdf8bd7a-a5c3-4b37-8fd0-201eeb31d073) |
| #19 | [MeSH descriptor **Education**, this term only](http://onlinelibrary.wiley.com/o/cochrane/searchHistory?mode=runquery&qnum=19) | 399 | [edit](http://onlinelibrary.wiley.com/search-web/cochrane/searchHistory?mode=editquery&qnum=19&searchKey=bdf8bd7a-a5c3-4b37-8fd0-201eeb31d073) | [delete](http://onlinelibrary.wiley.com/search-web/cochrane/searchHistory?mode=deletequery&qnum=19&uuid=bdf8bd7a-a5c3-4b37-8fd0-201eeb31d073&searchKey=bdf8bd7a-a5c3-4b37-8fd0-201eeb31d073) |
| #20 | [(train* or teach* or taught or learn*):ti,ab,kw](http://onlinelibrary.wiley.com/o/cochrane/searchHistory?mode=runquery&qnum=20) | 37100 | [edit](javascript:doPopup('/search-web/cochrane/searchHistory?mode=editquery&qnum=20&searchKey=bdf8bd7a-a5c3-4b37-8fd0-201eeb31d073', 400)) | [delete](http://onlinelibrary.wiley.com/search-web/cochrane/searchHistory?mode=deletequery&qnum=20&uuid=bdf8bd7a-a5c3-4b37-8fd0-201eeb31d073&searchKey=bdf8bd7a-a5c3-4b37-8fd0-201eeb31d073) |
| #21 | [MeSH descriptor **Pain** explode all trees](http://onlinelibrary.wiley.com/o/cochrane/searchHistory?mode=runquery&qnum=21) | 28635 | [edit](http://onlinelibrary.wiley.com/search-web/cochrane/searchHistory?mode=editquery&qnum=21&searchKey=bdf8bd7a-a5c3-4b37-8fd0-201eeb31d073) | [delete](http://onlinelibrary.wiley.com/search-web/cochrane/searchHistory?mode=deletequery&qnum=21&uuid=bdf8bd7a-a5c3-4b37-8fd0-201eeb31d073&searchKey=bdf8bd7a-a5c3-4b37-8fd0-201eeb31d073) |
| #22 | [MeSH descriptor **Pain Management**, this term only](http://onlinelibrary.wiley.com/o/cochrane/searchHistory?mode=runquery&qnum=22) | 1031 | [edit](http://onlinelibrary.wiley.com/search-web/cochrane/searchHistory?mode=editquery&qnum=22&searchKey=bdf8bd7a-a5c3-4b37-8fd0-201eeb31d073) | [delete](http://onlinelibrary.wiley.com/search-web/cochrane/searchHistory?mode=deletequery&qnum=22&uuid=bdf8bd7a-a5c3-4b37-8fd0-201eeb31d073&searchKey=bdf8bd7a-a5c3-4b37-8fd0-201eeb31d073) |
| #23 | [(pain*):ti,ab,kw](http://onlinelibrary.wiley.com/o/cochrane/searchHistory?mode=runquery&qnum=23) | 55736 | [edit](javascript:doPopup('/search-web/cochrane/searchHistory?mode=editquery&qnum=23&searchKey=bdf8bd7a-a5c3-4b37-8fd0-201eeb31d073', 400)) | [delete](http://onlinelibrary.wiley.com/search-web/cochrane/searchHistory?mode=deletequery&qnum=23&uuid=bdf8bd7a-a5c3-4b37-8fd0-201eeb31d073&searchKey=bdf8bd7a-a5c3-4b37-8fd0-201eeb31d073) |
| #24 | [(discomfort or irritation or success* or attempt* or error* or mistake* or refuse or refusal):ti,ab,kw](http://onlinelibrary.wiley.com/o/cochrane/searchHistory?mode=runquery&qnum=24) | 43938 | [edit](javascript:doPopup('/search-web/cochrane/searchHistory?mode=editquery&qnum=24&searchKey=bdf8bd7a-a5c3-4b37-8fd0-201eeb31d073', 400)) | [delete](http://onlinelibrary.wiley.com/search-web/cochrane/searchHistory?mode=deletequery&qnum=24&uuid=bdf8bd7a-a5c3-4b37-8fd0-201eeb31d073&searchKey=bdf8bd7a-a5c3-4b37-8fd0-201eeb31d073) |
| #25 | [MeSH descriptor **Risk** explode all trees](http://onlinelibrary.wiley.com/o/cochrane/searchHistory?mode=runquery&qnum=25) | 24499 | [edit](http://onlinelibrary.wiley.com/search-web/cochrane/searchHistory?mode=editquery&qnum=25&searchKey=bdf8bd7a-a5c3-4b37-8fd0-201eeb31d073) | [delete](http://onlinelibrary.wiley.com/search-web/cochrane/searchHistory?mode=deletequery&qnum=25&uuid=bdf8bd7a-a5c3-4b37-8fd0-201eeb31d073&searchKey=bdf8bd7a-a5c3-4b37-8fd0-201eeb31d073) |
| #26 | [MeSH descriptor **Emotions** explode all trees](http://onlinelibrary.wiley.com/o/cochrane/searchHistory?mode=runquery&qnum=26) | 9850 | [edit](http://onlinelibrary.wiley.com/search-web/cochrane/searchHistory?mode=editquery&qnum=26&searchKey=bdf8bd7a-a5c3-4b37-8fd0-201eeb31d073) | [delete](http://onlinelibrary.wiley.com/search-web/cochrane/searchHistory?mode=deletequery&qnum=26&uuid=bdf8bd7a-a5c3-4b37-8fd0-201eeb31d073&searchKey=bdf8bd7a-a5c3-4b37-8fd0-201eeb31d073) |
| #27 | [(anxiety or anxious* or concern* or worry or worried or fear* or phobia* or distress* or coping or trauma* or stress* or suffer* or experience):ti,ab,kw](http://onlinelibrary.wiley.com/o/cochrane/searchHistory?mode=runquery&qnum=27) | 92301 | [edit](javascript:doPopup('/search-web/cochrane/searchHistory?mode=editquery&qnum=27&searchKey=bdf8bd7a-a5c3-4b37-8fd0-201eeb31d073', 400)) | [delete](http://onlinelibrary.wiley.com/search-web/cochrane/searchHistory?mode=deletequery&qnum=27&uuid=bdf8bd7a-a5c3-4b37-8fd0-201eeb31d073&searchKey=bdf8bd7a-a5c3-4b37-8fd0-201eeb31d073) |
| #28 | [MeSH descriptor **Patient Acceptance of Health Care** explode all trees](http://onlinelibrary.wiley.com/o/cochrane/searchHistory?mode=runquery&qnum=28) | 16276 | [edit](http://onlinelibrary.wiley.com/search-web/cochrane/searchHistory?mode=editquery&qnum=28&searchKey=bdf8bd7a-a5c3-4b37-8fd0-201eeb31d073) | [delete](http://onlinelibrary.wiley.com/search-web/cochrane/searchHistory?mode=deletequery&qnum=28&uuid=bdf8bd7a-a5c3-4b37-8fd0-201eeb31d073&searchKey=bdf8bd7a-a5c3-4b37-8fd0-201eeb31d073) |
| #29 | [MeSH descriptor **Patient Satisfaction** explode all trees](http://onlinelibrary.wiley.com/o/cochrane/searchHistory?mode=runquery&qnum=29) | 7372 | [edit](http://onlinelibrary.wiley.com/search-web/cochrane/searchHistory?mode=editquery&qnum=29&searchKey=bdf8bd7a-a5c3-4b37-8fd0-201eeb31d073) | [delete](http://onlinelibrary.wiley.com/search-web/cochrane/searchHistory?mode=deletequery&qnum=29&uuid=bdf8bd7a-a5c3-4b37-8fd0-201eeb31d073&searchKey=bdf8bd7a-a5c3-4b37-8fd0-201eeb31d073) |
| #30 | [(patient Near/3 (preference or satisf*)):ti,ab,kw](http://onlinelibrary.wiley.com/o/cochrane/searchHistory?mode=runquery&qnum=30) | 11833 | [edit](javascript:doPopup('/search-web/cochrane/searchHistory?mode=editquery&qnum=30&searchKey=bdf8bd7a-a5c3-4b37-8fd0-201eeb31d073', 400)) | [delete](http://onlinelibrary.wiley.com/search-web/cochrane/searchHistory?mode=deletequery&qnum=30&uuid=bdf8bd7a-a5c3-4b37-8fd0-201eeb31d073&searchKey=bdf8bd7a-a5c3-4b37-8fd0-201eeb31d073) |
| #31 | [(quality Near/3 life):ti,ab,kw](http://onlinelibrary.wiley.com/o/cochrane/searchHistory?mode=runquery&qnum=31) | 23790 | [edit](javascript:doPopup('/search-web/cochrane/searchHistory?mode=editquery&qnum=31&searchKey=bdf8bd7a-a5c3-4b37-8fd0-201eeb31d073', 400)) | [delete](http://onlinelibrary.wiley.com/search-web/cochrane/searchHistory?mode=deletequery&qnum=31&uuid=bdf8bd7a-a5c3-4b37-8fd0-201eeb31d073&searchKey=bdf8bd7a-a5c3-4b37-8fd0-201eeb31d073) |
| #32 | [(#1 OR #2 OR #3 OR #4 OR #5 OR #6 OR #7 OR #8 OR #9 OR #10 OR #11 OR #12)](http://onlinelibrary.wiley.com/o/cochrane/searchHistory?mode=runquery&qnum=32) | 1526 | [edit](javascript:doPopup('/search-web/cochrane/searchHistory?mode=editquery&qnum=32&searchKey=bdf8bd7a-a5c3-4b37-8fd0-201eeb31d073', 400)) | [delete](http://onlinelibrary.wiley.com/search-web/cochrane/searchHistory?mode=deletequery&qnum=32&uuid=bdf8bd7a-a5c3-4b37-8fd0-201eeb31d073&searchKey=bdf8bd7a-a5c3-4b37-8fd0-201eeb31d073) |
| #33 | [(#13 OR #14 OR #15 OR #16 OR #17 OR #18 OR #19 OR #20 OR #21 OR #22 OR #23 OR #24 OR #25 OR #26 OR #27 OR #28 OR #29 OR #30 OR #31)](http://onlinelibrary.wiley.com/o/cochrane/searchHistory?mode=runquery&qnum=33) | 320837 | [edit](javascript:doPopup('/search-web/cochrane/searchHistory?mode=editquery&qnum=33&searchKey=bdf8bd7a-a5c3-4b37-8fd0-201eeb31d073', 400)) | [delete](http://onlinelibrary.wiley.com/search-web/cochrane/searchHistory?mode=deletequery&qnum=33&uuid=bdf8bd7a-a5c3-4b37-8fd0-201eeb31d073&searchKey=bdf8bd7a-a5c3-4b37-8fd0-201eeb31d073) |
| #34 | [(#32 AND #33), from 1990 to 2012](http://onlinelibrary.wiley.com/o/cochrane/searchHistory?mode=runquery&qnum=34) | 1059 | [edit](javascript:doPopup('/search-web/cochrane/searchHistory?mode=editquery&qnum=34&searchKey=bdf8bd7a-a5c3-4b37-8fd0-201eeb31d073', 400)) | [delete](http://onlinelibrary.wiley.com/search-web/cochrane/searchHistory?mode=deletequery&qnum=34&uuid=bdf8bd7a-a5c3-4b37-8fd0-201eeb31d073&searchKey=bdf8bd7a-a5c3-4b37-8fd0-201eeb31d073) |
| #35 | [(#1 OR #34)](http://onlinelibrary.wiley.com/o/cochrane/searchHistory?mode=runquery&qnum=35) | 1169 | [edit](javascript:doPopup('/search-web/cochrane/searchHistory?mode=editquery&qnum=35&searchKey=bdf8bd7a-a5c3-4b37-8fd0-201eeb31d073', 400)) | [delete](http://onlinelibrary.wiley.com/search-web/cochrane/searchHistory?mode=deletequery&qnum=35&uuid=bdf8bd7a-a5c3-4b37-8fd0-201eeb31d073&searchKey=bdf8bd7a-a5c3-4b37-8fd0-201eeb31d073) |
| #36 | [(#35 AND NOT #34)](http://onlinelibrary.wiley.com/o/cochrane/searchHistory?mode=runquery&qnum=36) | 110 | [edit](javascript:doPopup('/search-web/cochrane/searchHistory?mode=editquery&qnum=36&searchKey=bdf8bd7a-a5c3-4b37-8fd0-201eeb31d073', 400)) | [delete](http://onlinelibrary.wiley.com/search-web/cochrane/searchHistory?mode=deletequery&qnum=36&uuid=bdf8bd7a-a5c3-4b37-8fd0-201eeb31d073&searchKey=bdf8bd7a-a5c3-4b37-8fd0-201eeb31d073) |
| #37 | [(#34 OR #36)](http://onlinelibrary.wiley.com/o/cochrane/searchHistory?mode=runquery&qnum=37) | 1169 | [edit](javascript:doPopup('/search-web/cochrane/searchHistory?mode=editquery&qnum=37&searchKey=bdf8bd7a-a5c3-4b37-8fd0-201eeb31d073', 400)) | [delete](http://onlinelibrary.wiley.com/search-web/cochrane/searchHistory?mode=deletequery&qnum=37&uuid=bdf8bd7a-a5c3-4b37-8fd0-201eeb31d073&searchKey=bdf8bd7a-a5c3-4b37-8fd0-201eeb31d073) |

**12.**

**Database:** Cinahl

**Host:** Ebesco Host

**Data Parameters:**

**Date Searched:** Monday, March 12^th^ 2012

**Searcher:** Cooper

**Hits:**  179

**Strategy:**

(MH "Catheterization, Peripheral")

(((venous N3 Catheter*) AND peripheral))

PIVC

((venous N3 Cannula*))

(((vein* N3 (Catheter*)) AND (peripheral)))

((vein* N3 cannula*))

(((intravenous OR IV) N3 cannula*))

(((intravenous OR IV) N3 catheter*))

(((multiple OR repeat*) N3 (cannula* OR Catheter*)))

((cannula* OR cather*) N3 (puncture* OR insert* OR needle))

((Venipuncture OR venepuncture))

S1 OR S2 OR S3 OR S4 OR S5 OR S6 OR S7 OR S8 OR S9 OR S10 OR S11

(adverse N3 event*)

(complication* or infection* or prepar* or compliance or infiltration or extravasation or phlebitis or thrombophlebitis or (VIP) or train* or teach* or taught or learn* or pain* or discomfort or irritation or success* or attempt* or error* or mistake* or refuse or refusal or anxiety or anxious* or concern* or worry or worried or fear* or phobia* or distress* or coping or trauma* or stress* or suffer* or experience)

(patient N3 (preference or satisf*))

(quality N3 life)

S13 OR S14 OR S15 OR S16

S12 and S17

Limit S18 to Humans

Limit to S19 1990-2012

**Notes:** A server side de-duplication was conducted to remove Medline records.

**File Name:** Cinahl

Database: Prospero

Host:

Data Parameters:

Date Searched: Monday, March 12^th^ 2012

Searcher: Cooper

Hits: 4

Strategy:

Venous Cannulation n=0

Venous Catheter* n=4

<http://www.crd.york.ac.uk/prospero/display_record.asp?ID=CRD42011001685>

<http://www.crd.york.ac.uk/prospero/display_record.asp?ID=CRD42011001790>

<http://www.crd.york.ac.uk/prospero/display_record.asp?ID=CRD42012001952>

<http://www.crd.york.ac.uk/prospero/display_record.asp?ID=CRD42012001967>
