# Supplementary material for: First do no harm: pain relief for the peripheral venous cannulation of adults, a systematic review and network meta-analysis
Source: BMC Anesthesiol. 2016 Oct 1;16:81. doi: 10.1186/s12871-016-0252-8 (PMC5045592; doi:10.1186/s12871-016-0252-8)
Supplement: Supplementary file 2 — Local anaesthestics used in the studies included in the systematic review.docx Local anaesthetics used in the included studies. Summary local anaesthestics used in the included studies. (DOCX 17 kb) [file 12871_2016_252_MOESM2_ESM.docx]

| **Local Anaesthetic** | **Description** |
| --- | --- |
| Ametop® (S&N Healthcare) | **Gel**, tetracaine (amethocaine) 4%, 1.5-g tube. |
| Bupivacaine hydrochloride | **Injection,** anhydrous bupivacaine hydrochloride: 2.5 mg/mL (0.25%), 10 mL 5 mg/mL (0.5%), 10 mL |
| Chloroprocaine | **Injection,** 2% part of the family of esters of aminobenzoic acids eg. Tetracaine |
| EMLA® cream (AstraZeneca) | **Cream**, lidocaine 2.5%, prilocaine 2.5%: 5-g tube 30-g tube (surgical pack) 5 × 5-g tube with 12 occlusive dressings (premedication pack) |
| Ethyl Chloride | **spray,** a colourless, flammable gas that rapidly chills the skin producing mild local anaesthesia |
| Iiontocaine | Local anesthetic with vasoconstrictor, known by the brand name Numby®, administered via iontophoresis through the skin. It can numb up to 10 mm of skin as quick as 10 minutes. It is a 2% lidocaine, 0.01 mg/ml epinephrine solution. Manufactured by IOMED, Inc. Iontophoresis, also called electromotive drug administration (EMDA), is a technique using a small electric charge to deliver a medicine or other chemical through the skin |
| Lidocaine (ligocaine) | **Injection**, lidocaine hydrochloride: 5 mg/mL (0.5%), 10-mL amp 10 mg/mL (1%), 2-mL amp 5-mL amp = 28p, 10-mL amp 10-mL prefilled syringe 20-mL amp 20 mg/mL (2%), 2-mL amp 5-mL amp |
| Myolaxin ointment (Geno Pharmaceuticals) | **Ointment,** oleoresin capsicum equivalent to capsicum 0.075% , methylsalicylate IP 20% , mentol IP 10% , camphor USP 5% and eucalyptus oil IP 5% . Previously used in the treatment of chronic non-eurogenic and myofascial pain. |
| Sodium chloride with preservative | **Injection,** saline plus 0.9% benzyl alcohol |
| Sodium chloride | **Injection,** saline |
| Tetracaine (amethocaine) | **Cream,** 5% |
