# Supplementary material for: First do no harm: pain relief for the peripheral venous cannulation of adults, a systematic review and network meta-analysis
Source: BMC Anesthesiol. 2016 Oct 1;16:81. doi: 10.1186/s12871-016-0252-8 (PMC5045592; doi:10.1186/s12871-016-0252-8)
Supplement: Supplementary file 7 — Probability of being the most effective.docx Probability of effectiveness. Analysis of probability of most effective drug. (DOCX 13 kb) [file 12871_2016_252_MOESM7_ESM.docx]

| **Local anaesthetic** | **Probability** |
| --- | --- |
| Lidocaine 2% | 43.9% |
| Lidocaine + methylparaben | 17.3% |
| Iontocaine | 15.2% |
| Rapydan patch | 10.9% |
| Bupivacaine | 7.3% |
| Lidocaine + NaCHO3 | 3.7% |
| Buffered lidocaine 1% | <1% |
| Chloroprocaine | <1% |
| Diclofenac patch | <1% |
| Dichlorotetrafluoroethane spray | <1% |
| Lidocaine 1% | <1% |
| Ethyl chloride spray | <1% |
| Ametop cream | <1% |
| Buffered saline | <1% |
| Iontocaine + placebo cream | <1% |
| EMLA cream | 0% |
| Saline | 0% |
| Placebo cream | 0% |
| Placebo patch | 0% |
| No treatment | 0% |
