# Supplementary material for: First do no harm: pain relief for the peripheral venous cannulation of adults, a systematic review and network meta-analysis
Source: BMC Anesthesiol. 2016 Oct 1;16:81. doi: 10.1186/s12871-016-0252-8 (PMC5045592; doi:10.1186/s12871-016-0252-8)
Supplement: Supplementary file 8 — NMA other results plots.docx Results plots from the NMA. Other results plots from the NMA. (DOCX 108 kb) [file 12871_2016_252_MOESM8_ESM.docx]

Forest plots of the pooled estimates using direct and indirect evidence

Comparator treatment is buffered saline

Comparator treatment is saline

Comparator treatment is placebo cream

Comparator treatment is buffered lidocaine 1%

Comparator treatment is lidocaine + NaCHO3

Comparator treatment is lidocaine + methylparaben

Comparator treatment is chlorprocaine

Comparator treatment is bupivacaine

Comparator treatment is iontocaine

Comparator treatment is diclofenac patch

Comparator treatment is dichlorotetrafluoroethane spray

Comparator treatment is placebo patch

Comparator treatment is Rapydan patch

Comparator treatment is ethyl chloride spray
