# Supplementary material for: First do no harm: pain relief for the peripheral venous cannulation of adults, a systematic review and network meta-analysis
Source: BMC Anesthesiol. 2016 Oct 1;16:81. doi: 10.1186/s12871-016-0252-8 (PMC5045592; doi:10.1186/s12871-016-0252-8)

Direct and Indirect evidence

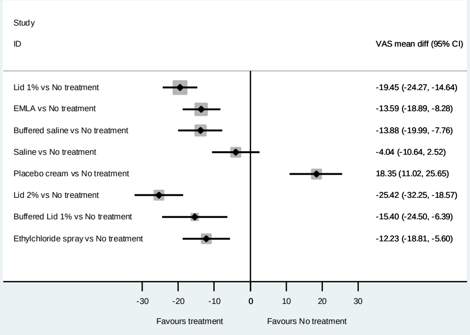

Direct evidence only

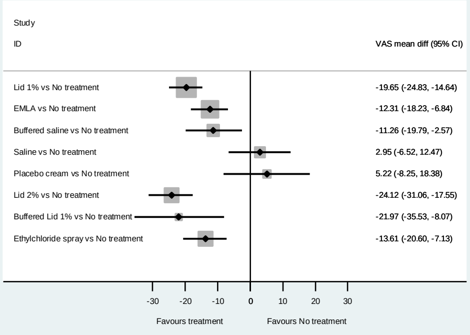

Supplement: Supplementary file 5 [file 12871_2016_252_MOESM10_ESM.docx]
