# Supplementary material for: First do no harm: pain relief for the peripheral venous cannulation of adults, a systematic review and network meta-analysis
Source: BMC Anesthesiol. 2016 Oct 1;16:81. doi: 10.1186/s12871-016-0252-8 (PMC5045592; doi:10.1186/s12871-016-0252-8)
Supplement: Supplementary file 6 [file 12871_2016_252_MOESM3_ESM.docx]

Study/Author Year (Country)	Design	N	Participants	PVC site	Premed	Intervention group	Control group	Gauge	Outcomes	Exclusion criteria
Agarwal et al 2006 (India)	Randomised, double-blind, placebo-controlled	72	Adults (16-60 years) undergoing elective surgery (lasting <1 hour); ASA status I or II	dorsum hand	NR	Diclofenac-Buttock (diclofenac diethylamine BP 100 mg, absorption 50cm2), 60 mins N=24<br><br>Diclofenac-Hand (diclofenac diethylamine BP 100 mg, absorption 50cm2), 60 mins N=24	Placebo patch, hand and buttock, 60 mins N=24	18-G	Pain (non-graduated 10 cm VAS)	Patients taking NSAIDs regularly, known allergy or sensitivity to NSAIDs, communication problems, and abnormal skin conditions
Agarwal et al 2007 (India)	Randomised, double-blind	60	Adults (15-60 years); ASA status I or II; either gender; undergoing laparoscopic cholecystectomy	Dorsum hand	Yes, lorazepam 2mg	Group 1: EMLA cream N=30	Group 2: Myolaxin ointment N=30	18-G	Pain (10-point VAS); adverse events (local blanching, erythema, swelling at site of VC)	Patients with peripheral neuropathy; long-term consumption of analgesics; abnormal skin condition at the site of venous puncture; allergy or any other side effect secondary to application of cream; and mental disorder
Agarwal et al 2007a (India)	Randomised, placebo-controlled, double-blind	450	Adults (18-60 years); either sex; ASA status I or II scheduled for endo-urological procedures	Dorsum hand	NR	Group 2: EMLA cream (2g/10cm2) N=150<br><br>Group 3: Transdermal diclofenac patch (diclofenac diethylamine BP 100 mg, absorption area of 50cm2) N=150	Group 1: Placebo N=150	NR	Pain (10-point VAS)	Known sensitvity to local anaesthetics; allergy to NSAIDs; skin lesions at site of application; skin infection; history of analgesic intake (previous 24 hours); inability to communicate
Armstong et al 1990 (UK)	Randomised controlled trial	120	Unpremedicated adult female patients undergoing minor gynaecological day-case surgery	Dorsum hand	No	0.2 ml lidocaine intradermally 25G needle, 30 secs<br><br>Ethyl chloride sprayed from 20cm for 10 seconds, VC immediately after	No local anaesthetic	20-G	***Investigator:*** Difficulties of cannulation (vein visibility) before and after skin anaesthesia and ease of cannula insertion using a five-point scale; ***Patient:*** pain of anaesthetic application; of catheter insertion; and the skin pain after insertion using a 10cm VAS	NR
Ashburn et al 1997 (USA)	Randomised, double-blind, placebo-controlled	43	Adults requiring IV cannulation before scheduled eye surgery	NR	NR	Iontophoresis lidocaine HCl 2% with epinephrine 1:100,000 N=21	Placebo N=20	20-G	Pain (***patient assessed -*** VAS; evaluation delivery eliminate pain during the IV insertion procedure and would use method again; ***investigator assessed -*** whether iontophoretic drug delivery provided adequate anaesthesia), adverse events	NR
Beck et al 2011 (USA)	Randomised, double-blind control trial	120	Adult patients (18 years-plus) scheduled for an elective surgical procedure; competent to make decisions; able to read and speak English	Dorsum hand	NR	1% lidocaine (N=30)<br><br>0.5% bupivacaine (N=30)<br><br>2% chloroprocaine (N=30)<br><br>1% lidocaine and 8.4% sodium bicarbonate (N=30)		18-G	Pain (injection of the local anaesthetic injection, and IV catheter insertion) VAS (100 mm); number of attempts	Patients with a previous allergic reaction to any local anaesthetic

Study/Author Year (Country)	Design	N	Participants	PVC site	Premed	Intervention group	Control group	Gauge	Outcomes	Exclusion criteria
Biro et al. 1997 (Switzerland)	Randomised controlled trial	150	Adults (16 and 65 years); ASA status I or II; unselected surgical patients	Dorsum hand	No	EMLA cream 2g (applied to dorsal side of hand), 60 mins N=28<br><br>Ethyl chloride spray (applied from height 25 cm until a layer of frosting had developed, immediately followed by insertion of the cannula) N=30<br><br>[illegible]	No local anaesthetic N=30	18-G	Pain (VAS 100 mm); during cannulation heart rate, blood pressure and verbal and motordefence reactions were measured	Emergency surgery; allergies to local anaesthetics of amide type; dermal diseases; neuropathy; systemic analgesia administered within previous 12 hours
Brown and Larson 1999 (USA)	Controlled trial, convenience sample	100	Adults alert and oriented, spoke English, needed to have a peripheral IV inserted,	NR	NR	0.1cc 1% lidocaine hydrochloride (intradermal) N=50	Placebo N=50		Pain (anticipated and actual, 0 to 10 number and a face scale) number of attempts at insertion, time required for insertion	Not allergic to lidocaine
Brown 2003 (USA)	Survey	188	Convenience sample of adults (≥18 years), alert and oriented, English speakers, PVC in place at the time of the interview.	NR	NR	Lidocaine administered prior to current or previous PVC N=101	No local anaesthetic N=79	NR	Patient preferences for lidocaine injection prior to PVC	NR
Burke et al 2011 (USA)	Randomised, double-blind, parallel-design, controlled trial	148	Adults (18-80 years); read, speak and understand English; physician's order for a peripheral IV line; ability to rate and express pain level	Forearm, hand and anticubital sites	NR	Intradermal buffered lignocaine (5 ml of 8.4% sodium bicarbonate + 50 ml vial of 1% lidocaine to fill 6 30-G tuberculin syringes with 0.2ml of solution) N=74	Intradermal bacteriostatic normal saline (6 30-G tuberculin syringes of 0.2 ml of bacteriostatic normal saline) N=74	16-G, 18-G, 20-G, 22-G	Pain (verbal numeric rating scale 0 = no pain and 10 = worst pain imagineable)	Patients with a history of lidocaine allergy and those who'd taken pain medication within the previous four hours
Campbell-Jones 2010 (USA)	Randomised, double-blind	60	Adults (≥18 years); ability to express and rate levels of pain; no prior pain medication; no known allergy to lidocaine-related medications; requirement for peripheral catheter insertion	Forearm	NR	Lidocaine hydrochloride (1%) N=20<br><br>Normal saline with preservatives N=22	Normal saline N=18	NR	Pain (Wong-Baker FACES a 0 - 10 point pain rating scale)	More than one attempt to insert the IV catheter
Celik et al. 2011 (Turkey)	controlled cross-over study	41	Adult haemodialysis patients	Arm	NR	1% lidocaine [N=84]<br>1% buffered lidocaine [N=85]<br>bacteriostatic saline [N=83]	control: no treatment	18-G	Pain VAS (100 mm)	allergic to local anaesthetics, damaged skine , concomitant use of analgesia within previous 24 h, previous use of EMLA

Study/Author Year (Country)	Design	N	Participants	PVC site	Premed	Intervention group	Control group	Gauge	Outcomes	Exclusion criteria
Deguzman et al. 2012 (USA)	Randomised, double-blind control trial	376	Adults in preoperative care unit (18 yrs and older). English speakers and writers, able to rate pain	Hand, arm or anticubital fossa	No	1% buffered lidocaine (2ml bicarbonate of soda for 22mL of 1% lidocaine)	Bacteriostatic saline (9mg sodium chloride, 0.9% benzyl alcohol)	18 G, 20-G or 22-G	Pain measured on a NRS (0 -10 scale). Each participant rated either the pain of anaesthetic or the pain of PVC	Allergic to lidocaine, unsuccessful first attempt at IV, needle phobic, anxious, panic attack, neuropathy or cognitive impairment
Deshpande and Jain 2010 (India)	Randomised, double-blind	200	Adults (16-60 years); ASA status I and II; undergoing elective surgery	Dorsum hand	NR	EMLA cream (2 g/10cm2) (60-70 min) N=100	Diclofenac Transdermal Patch (100mg/50cm2) (60-70 min) N=100	18-G	Pain (VAS)	Known allergy to NSAIDs; skin imperfection at site of application; prior analgesic intake within previous 24 hours; patients with inability to communicate; history of previous use of EMLA or diclofenac patch for IV cannulation
Dickey 1988 (USA)	Controlled trial, convenience sample	31	Preoperative day surgery adult patients (18-65 years) requiring IV catheter insertion	Lateral cephalic vein, medial to the wrist; back of the hand; dorsal forearm; ventral forearm	No	Lidocaine 0.1 cc, (N=11)	Group 1 = placebo injection (N=10); Group 2 = no pretreatment (N=10)	18-G	Pain (pain-scale questionnaire); number of insertion attempts; degree of difficulty	Known or suspected allergies to local anaesthetics; patients premedicated with narcotic analgesics, and those with acute pain
Ganter-Ritz et al. 2012 (USA)	Randomised, double-blind control trial	256	Adult same day surgical patients, 18 years or over, visible or palpable vein	Hand or arm	NR	1% lidocaine [N=84]<br>1% buffered lidocaine [N=85]<br>bacteriostatic saline [N=83]		20-G	a) remembered pain from past PVC 0-10, b)anxiety about immendent PVC, 0-4 c) pain of current PVC insertion 0-10 on 10cm VAS	Non English speaker, allergy to interventions and inability to complete patient rating scale
Gunawardene et al 1990 (UK)	Randomised, double-blind	100		Dorsum hand	No	Group 1: EMLA (2.5g)+glyceryl trinitrate (1-2 mg), 60 mins N=25<br><br>Group 3: EMLA only, 60 mins N=25<br><br>Group 4: Glyceryl trinitrate only, 60 mins N=25	Group 2: Placebo (KY Jelly), 60 mins N=25	23-G	Pain and ease of venepuncture at induction of anaesthesia; pain post operation; all verbal	NR
Gupta et al 2013 (India)	Randomised controlled trial	120	Elective surgical patients [15-60 years] cannulated at the first attempt	Dorsum hand	Yes 2 mg lorazepam	Group 1 EMLA [N=30]<br>Group 2 Myolaxin [N=30]<br>Group 3 EMLA = Myolaxin [N=30]	lubricant cream [N=30]	18-G	Incidence of 0 pain score on 10 cm VAS	peripheral neuropathy, taking analgesics, abnormal skin, blindness or communication problems

Study/Author Year (Country)	Design	N	Participants	PVC site	Premed	Intervention group	Control group	Gauge	Outcomes	Exclusion criteria
Harasawa et al 1993 (Japan)	Randomised controlled trial	26	Unpremedicated adult patients undergoing elective surgery and subjected to insertion of an IV cannula	Dorsum hand or wrist	No	Group A: Lidocaine patch (15 mg lidocaine) - 15 mins (N=12)	Group B: Lidocaine patch (15 mg lidocaine) - 30 mins (N=14)	16-G or 18-G	Pain (VAS, 100 mm)<br><br>Arterial plasma concentration of lidocaine by homogenous enzyme immunoassay 15 min after patch application (n/N = 7/26)	
Hattula et al. 2002 (USA)	Randomised controlled trial	33	Adults in a 35 bed unit	Hand or forearm	NR	Buffered lidocaine 1% Normal saline buffered with 0.9% benzyl alcohol	No treatment	18-20G	VAS (range of scale not reported, assumed to be 0-10 on the basis of the results)	Mental impairment, inability to speak English, inury to both upper extremities, allegic to benzyl alcohol or lidocaine, in acute distress or severe pain.
Hussey et al 1997 (USA)	Randomised, double blind, control trial	99	Unpremedicated adult patients (18 years-plus) scheduled for elective surgical procedures requiring IV therapy; alert and oriented; not in acute pain; English as primary language	Hand 90%, wrist 7% forearm 2% antecubital fossa 1%	No	Group 1: Lidocaine hydrochloride intradermal injection, 15-30 secs<br><br>Group 2: Sodium chloride intradermal injection, 15-30 secs	Group 3: No local anaesthetic, 15-30 secs	18-G	Pain (VAS 100 mm)	Those with known allergies to local anaesthetic agents
Kitamoto et al 1992 (Japan)	Randomised, placebo-controlled, double-blind, cross-over design	16	Adult haemodialysis patients	Forearm	NR	Group 3: 10% lidocaine gel, 60 mins N=16	Group 3: Placebo gel, 60 mins N=16	18-G	Pain (scale 5 points; o indicates no pain and 4 extreme pain); blood pressure	NR
Lysakowski et al 2003 (USA)	Randomised controlled trial	400	Surgical adult inpatients	Dorsum hand	Y (30 mins)a	Jet, 0.5 ml saline (N=100)<br><br>Jet, 0.5 ml with 1% lidocaine (N=100)<br><br>Jet, 0.5 ml 2% lidocaine (N=100)	No treatment (N=100)	18-G	Pain (numerical verbal scale [NVS 0-10]<br><br>Cost - annual cost of PVC via Jet, cost incurred in generating one gainer ICER	Preoperative analgesic treatment, allergy to local anaesthetics, neuropathies, and pregnancy
Maddi et al. 1990 (USA)	Randomised double-blind placebo-controlled trial	75 (own controls)	Adult patients scheduled for orthopaedic or cardiac surgery	Dorsum hand	Yes	EMLA cream<br>group1: N=30, 30 min wait for PVC,<br>group 2: N=20, 45 min wait for PVC,<br>group 3: N=25, 60 min wait for PVC	Placebo cream, miglyol oil, visually identical to the EMLA cream<br>group1: N=30, 30 min wait for PVC,<br>group 2: N=20, 45 min wait for PVC,<br>group 3: N=25, 60 min wait for PVC	16-G	verbal categorisation of pain as either: none, mild, moderate or severe. Blood pressure and pulse before and after cannulation.	NR
Miller et al 2001 (USA)	Randomised controlled trial	30	Adults (≥18 years); ASA status I, II or III; undergoing outpatient or same-day surgery	Dorsum hand or forearm	NR	EMLA cream 2.5 mg N=10<br><br>1 ml Iontocaine N=10	SC injection of 0.3 to 0.5 ml of 1% lidocaine N=10	18-G	Pain VAS 10 point scale	Known allergy to amide lical anaesthetics; presence of a pacemaker or similar; use of monoamine oxidase inhibitors or tricyclic antidepressants; rash or impaired skin integrity at intended insertion site; IV catheter insertion requiring more than one attempt

Study/Author Year (Country)	Design	N	Participants	PVC site	Premed	Intervention group	Control group	Gauge	Outcomes	Exclusion criteria
Molodecka et al 1994 (UK)	Randomised, double-blind	120	Adult women; ASA status I or II; unpremedicated; undergoing day case gynaecological procedures	Dorsum hand	NR	5% EMLA cream 2.5 g, 30 mins N=30<br><br>5% EMLA cream 2.5 g, 60 mins N=30	Amethocaine cream 1 g (5% w/w), 30 mins N=30<br><br>Amethocaine cream 1 g (5% w/w), 60 mins N=30		Pain (***Investigator and patient:*** VAS 100 mm and 4-point rank score)	Known or suspected hypersensitivity to local anaesthetics
Moppet et al 2004 (UK)	Randomised, double-blind, controlled	28	Adults patients (19-77 years) ASA status I-III; undergoing elective ear, nose and throat surgery	Dorsum hand	NR	10 min 2 mA iontophoresis with lidocaine 4% and epinephrine (1 in 50,000) + sham cream - 60 mins	EMLA cream - 60 mins + sham iontophoresis (N=	18-G	Pain (10-point verbal rating scale); time to complete cannulation; degree of difficulty	Expected difficult venous access, disease or brokoen skin on hands and allergy to EMLA lidocaine or any part of the iontophoresis equipment
O'Connor and Tomlinson 1995 (UK)	Randomised, placebo-controlled, double-blind	42	Unpremedicated adults; ASA status I and II; undergoing day case procedures under general anaesthesia	Dorsum hand	No	Amethocaine gel (1 g 4% w/w amethocaine), 40 mins N=21	Placebo gel 1g, 40 mins N=21	20-G	Pain (three-point verbal rating scale [severity 0, 1, or 2]; VAS (100 mm); heart rate, blood pressure	Known or suspected hypersenstivity to local anaesthetics, broken skin, used analgesic preparations within the previous 24 h; women not using effective contraceptive protection; less than 18 years of age
Patterson et al 2000 (USA)	Randomised controlled trial	238	Adults scheduled for surgery (military teaching hospital) who required intravenous access	Dorsum hand	NR	Lidocaine 2.5% prilocaine 2.5% topical cream, 60-240 mins N=49<br>Dichlorotetrafluoroethane spray (applied from height of 15cm for 10 secs) N=63<br><br>0.1ml of 0.5% lidocaine with methylparaben N=65	0.1 ml of isotonic sodium chloride solution with 0.9% benzyl alcohol SC N=61	NR	Pain - 7-point verbal descriptor scale; and, VAS (100 mm)<br><br>Cost - unit dose<br><br>Convenience - ease of use or operation survey	NR
Ravishankar et al 2012 (UK)	Randomised, double-blind, controlled	100	Adults (18-65 years); ASA status I or II; both sexes; listed for day case surgery	Dorsum hand	NR	Rapydan patch (7% lidocaine and 7% tetracaine), 45 mins N=50	Ametop gel (4% tetracaine), 45 mins N=50	18-G	Pain (VAS 100 mm and descriptive pain [no pain, pressure, dull pain, sharp pain)<br><br>Adverse events: none reported	Pregnant or lactating women; known history of methemoglobinaemia, anaphylaxis or drug allergy; patient k=taking anti-epileptics or analgesic medication
Saxena et al 2004 (India)	Randomised, double-blind	60	Adult patients (15-70 years); ASA status I and II; scheduled for elective surgery	Dorsum hand	NR	Group 1: EMLA cream<br><br>Group 2: Lignocaine gel	Group 3: Vaseline jelly (placebo)	18-G	Pain (VAS 100 mm); change in pulse rate; change in blood pressure (SBP and DBP);	Patients with a history of allergy or hypersensitivity to any local anaesthetic
Selby and Bowles 1995 (UK)	Randomised, controlled trial; four-arm	160	Unpremedicated adult women; ASA status I or II; requiring the insertion of an IV cannula for administration of a general anaesthetic	Dorsum hand	No	EMLA group: EMLA cream (0.2 ml), 5 mins N=40<br><br>Spray group: Ethyl chloride sprayed over vein for 10 secs from a height of 20 cm and cannulation performed immedicately N=40<br><br>Lignocaine group: Lignocaine (0.2 ml of 1% lignocaine) injected SC, 30 secs N=40	No local anaesthetic N=40	20-G	Pain (VAS) on application of the local anaesthetic; at insertion of the IV cannula; and from the cannula one minute after insertion	Allergies to local anaesthetics, or analgesic medication taken within the previous 4 hours

Study/Author Year (Country)	Design	N	Participants	PVC site	Premed	Intervention group	Control group	Gauge	Outcomes	Exclusion criteria
Smith et al 2002 (USA)	Quasi-experimental	40	Unpremedicated adults undergoing ophthalmic surgery	Dorsum hand	No	EMLA cream 2.5 g, 5 mins N=20	Placebo (hypoallergenci hand cream) cream, 5 mins N=20	20-G	Pain (verbal numerical scale - 10 point scale used to report pain perception)	Patients needing emergency surgical procedures or with a known history of sensitivity to local anaesthetics of the amide type or to any other component of the amide type or to any other component of the product. Patients who received pre-operative medicines (narcotics or benzodiazepines). patients taking sulfonamides, nitroglyvcerin, and phenytoin.
Steinbrook et al 1993 (USA)	Randomised, double-blind study	184	Unpremedicated adult patients requiring insertion of an IV catheter prior to surgery	hand, wrist, forearm or anticubital fossa	No	1% lidocaine hydrochloride + 0.1 mEq/ml sodium bicarbonate (N=89)	1% lidocaine hydrochloride (N=95 patients)	NR	Pain (VAS, 100mm); location of infiltration and catheterisation; catheter size	NR
Vaghadia et al 1997 (Canada)	Randomised, double-blind, placebo-controlled, parallel group	50	Adult patients scheduled for outpatient surgery requiring preoperative venous cannulation age 18 years-plus and sufficient understanding of the VAS	Dorsum hand	No	EMLA patch (1 g of a 5% eutectic emulsion of lidocaine and prilocaine (N=24)	Placebo patch (N=26)	20-G	Pain (VAS, 100 mm); vaso-vagal reactions - patient and investigator assessed; ease of cannula insertion; blood pressure - investigator assessed; willingness to purchase in future - patient assessed	Previous use of EMLA for IV cannulation, allergy or sensitivity to amide local anaesthetics, use of a sedative or analgesci drug 12 hours before venous cannulation, participation in a clinical trial in the previous two weeks, and active dermatitis or open wound at the patch application site
Windle et al. 2006	Randomised controlled trial	197	Adults (≥18 years), able to read and write English, IV insertion in upper extremity	Forearm or back of hand	NR	Lidocaine 1% injection BNS 0.9% with benzyl alcohol injection	no treatment	18-G or 20-G	Pain of local anaesthetic Pain of IV insertion VAS (100 mm)	neuropathy and/or needle phobia, reanl aptietns, more than one attempt at IV insertion
Yeoh et al. 2012 (Malaysia)	Randomised controlled trial	80	Surgical adult inpatients [18-65 years]	Dorsum hand	No	Group 1 EMLA 5% [n=39] Group 2 Amethocaine 4% [N=41]		18-G	Pain 100mm VAS Ease of cannulation - 4 point scale	Concurrent analgesics or allergic to local anaesthetics, pregnant or breastfeeding women , diabetics with peripheral neuropathy or those with poorly visible veins

NR: not reported, NA: not applicable, VAS: visual analogue scale, VRS: verbal rating scale, ASA: American Society of Anesthesiologists, ICER: incremental cost-effectiveness ratio
