# Supplementary material for: First do no harm: pain relief for the peripheral venous cannulation of adults, a systematic review and network meta-analysis
Source: BMC Anesthesiol. 2016 Oct 1;16:81. doi: 10.1186/s12871-016-0252-8 (PMC5045592; doi:10.1186/s12871-016-0252-8)
Supplement: Supplementary file 7 [file 12871_2016_252_MOESM4_ESM.docx]

Section/Topic	Item No	Checklist item	1	2	3	4	5	6	7	8	9	10	11	12	13	14	15	16	17	18
**Title and abstract**																				
	1a	Identification as a randomised trial in the title	NR	NR	NR	NR	NR	NR	NR	NR	✓	NR	NR	NR	NR	NR	NR	✓	NR	NR
	1b	Structured summary of trial design, methods, results, and conclusions (for specific guidance see CONSORT for abstracts)	P	✓	NR	NR	NR	NR	P	NR	NR	P	P	P	NR	NR	NR	✓	NR	NR
**Introduction**																				
Background and objectives	2a	Scientific background and explanation of rationale	✓	✓	✓	✓	✓	P	✓	✓	✓	✓	✓	✓	✓	✓	P	✓	✓	✓
	2b	Specific objectives or hypotheses	✓	✓	✓	✓	✓	P	✓	✓	✓	✓	✓	✓	✓	✓	P	✓	✓	✓
**Methods**																				
Trial design	3a	Description of trial design (such as parallel, factorial) including allocation ratio	✓	✓	✓	✓	✓	✓	✓	✓	✓	✓	✓	✓	✓	✓	P	✓	✓	P
	3b	Important changes to methods after trial commencement (such as eligibility criteria), with reasons	NR	NR	NR	NR	NR	NR	NR	NR	NR	NR	NR	NR	NR	NR	NR	NR	NR	NR
Participants	4a	Eligibility criteria for participants	✓	✓	✓	✓	✓	✓	✓	✓	✓	✓	✓	✓	✓	✓	P	✓	✓	✓
	4b	Settings and locations where the data were collected	✓	NR	NR	NR	NR	NR	✓	✓	NR	NR	NR	NR	NR	NR	NR	P	NR	P
Interventions	5	The interventions for each group with sufficient details to allow replication, including how and when they were actually administered	✓	✓	✓	✓	✓	✓	✓	✓	✓	✓	✓	✓	✓	✓	P	✓	✓	✓
Outcomes	6a	Completely defined pre-specified primary and secondary outcome measures, including how and when they were assessed	✓	✓	✓	✓	✓	✓	✓	✓	✓	✓	✓	✓	✓	✓	✓	✓	✓	✓
	6b	Any changes to trial outcomes after the trial commenced, with reasons	NR	NR	NR	NR	NR	NR	NR	NR	NR	NR	NR	NR	NR	NR	NR	NR	NR	NR
Sample size	7a	How sample size was determined	✓	✓	NR	NR	NR	NR	✓	P	✓	✓	✓	NR	✓	NR	NR	NR	NR	NR
Harms	7b	When applicable, explanation of any interim analyses and stopping guidelines	NR	NR	NR	NR	NR	NR	NR	NR	NR	NR	NR	NR	NR	NR	NR	NR	NR	NR
**Randomisation**																				
Sequence generation	8a	Method used to generate the random allocation sequence	✓	✓	NR	NR	NR	NR	✓	✓	✓	NR	✓	NR	✓	NR	NR	NR	NR	NR
	8b	Type of randomisation; details of any restriction (such as blocking and block size)	✓	✓	NR	NR	NR	NR	✓	✓	✓	P	P	NR	✓	NR	NR	NR	NR	NR
Allocation concealment mechanism	9	Mechanism used to implement the random allocation sequence (such as sequentially numbered containers), describing any steps taken to conceal the sequence until interventions were assigned	✓	NR	NR	NR	NR	NR	✓	✓	✓	NR	NR	NR	✓	NR	NR	NR	NR	NR
Implementation	10	Who generated the random allocation sequence, who enrolled participants, and who assigned participants to interventions	✓	NR	NR	NR	NR	NR	✓	✓	NR	NR	NR	NR	✓	NR	NR	NR	NR	NR
Blinding	11a	If done, who was blinded after assignment to interventions (for example, participants, care providers, those assessing outcomes) and how	✓	NR	NR	NR	NR	NR	✓	✓	✓	NR	NR	NR	✓	NR	NR	NR	✓	NR
	11b	If relevant, description of the similarity of interventions	✓	NR	NR	NR	NR	NR	✓	NR	NR	✓	NR	NR	NR	NR	NR	✓	NR	NR
Statistical methods	12a	Statistical methods used to compare groups for primary and secondary outcomes	✓	✓	✓	✓	✓	P	✓	✓	✓	✓	✓	✓	✓	✓	✓	✓	✓	✓
	12b	Methods for additional analyses, such as subgroup analyses and adjusted analyses	NA	NA	NA	NA	NA	NA	NA	NA	✓	NA	NA	NA	NA	NA	NA	NA	NA	NA

Section/Topic	Item No	Checklist item	1	2	3	4	5	6	7	8	9	10	11	12	13	14	15	16	17	18
**Results**																				
Participant flow (a diagram is strongly recommended)	13a	For each group, the numbers of participants who were randomly assigned, received intended treatment, and were analysed for the primary outcome	NR	NR	NR	NR	NR	NR	NR	NR	P	NR	NR	NR	NR	NR	NR	NR	NR	NR
	13b	For each group, losses and exclusions after randomisation, together with reasons	✓	✓	NR	NR	NR	NR	P	NR	✓	NR	NR	NR	NR	P	NR	NR	NR	NR
Recruitment	14a	Dates defining the periods of recruitment and follow-up	NR	NR	NR	NR	NR	NR	NR	NR	NR	NR	NR	NR	NR	NR	NR	NR	NR	NR
	14b	Why the trial ended or was stopped	NA	NA	NA	NA	NA	NA	NA	NA	NA	NA	NA	NA	NA	NA	NA	NA	NA	NR
Baseline data	15	A table showing baseline demographic and clinical characteristics for each group	✓	NR	✓	✓	NR	NR	✓	P	NR	✓	✓	NR	NR	✓	✓	✓	NR	NR
Numbers analysed	16	For each group, number of participants (denominator) included in each analysis and whether the analysis was by original assigned groups	P	NR	P	P	NR	P	✓	P	✓	NR	P	NR	NR	P	P	✓	NR	NR
Outcomes and estimation	17a	For each primary and secondary outcome, results for each group, and the estimated effect size and its precision (such as 95% confidence interval)	P	P	✓	✓	✓	✓	✓	✓	✓	✓	✓	P	P	P	✓	✓	✓	✓
	17b	For binary outcomes, presentation of both absolute and relative effect sizes is recommended	NA	NA	NA	NA	NA	NA	NA	NA	NA	NA	NA	NA	NA	NA	NA	NA	NA	NA
Ancillary analyses	18	Results of any other analyses performed, including subgroup analyses and adjusted analyses, distinguishing pre-specified from exploratory	NA	NA	NA	NA	NA	NA	NA	NA	✓	✓	NR	NA	NA	NA	NA	NA	NA	NA
Harms	19	All important harms or unintended effects in each group (for specific guidance see CONSORT for harms)	P	✓	✓	P	P	P	NR	NR	✓	NR	P	✓	P	P	✓	✓	NR	✓
**Discussion**																				
Limitations	20	Trial limitations, addressing sources of potential bias, imprecision, and, if relevant, multiplicity of analyses	P	NR	NR	NR	NR	NR	✓	✓	✓	NR	NR	NR	✓	NR	NR	NR	NR	NR
Generalisability	21	Generalisability (internal validity, applicability) of the trial findings	✓	✓	✓	✓	✓	✓	✓	✓	✓	✓	✓	✓	✓	✓	P	✓	NR	NR
Interpretation	22	Interpretation consistent with results, balancing benefits and harms, and considering other relevant evidence	✓	✓	✓	✓	✓	✓	✓	✓	✓	✓	✓	✓	✓	✓	P	✓	✓	✓
**Other information**																				
Registration	23	Registration number and name of trial registry	NR	NR	NR	NR	NR	NR	NR	NR	NR	NR	NR	NR	NR	NR	NR	NR	NR	NR
Protocol	24	Where the full trial protocol can be accessed, if available	NR	NR	NR	NR	NR	NR	NR	NR	NR	NR	NR	NR	NR	NR	NR	NR	NR	NR
Funding	25	Sources of funding and other support (such as supply of drugs), role of funders	✓	NR	✓	✓	P	P	NR	NR	✓	NR	✓	✓	NR	NR	NR	NR	NR	NR

1. Ravishankar et al 2012 (UK); 2. Moppet et al 2004 (UK); 3. Selby et al 1995 (UK); 4. Molodecka et al 1994 (UK); 5. Armstrong et al 1990 (UK); 6. Gunawardene et al 1990 (UK); 7. Beck et al 2011 (USA); 8. Burke et al 2011 (USA); 9. Campbell-Jones et al 2010 (USA); 10. Lysakowksi et al 2003 (USA); 11. Miller et al 2001 (USA); 12. Patterson et al 2000 (USA); 13. Biro et al 1997 (Switzerland); 14. Hussey et al 1997 (USA); 15. Harasawa et al 1993 (Japan); 16 Celik et al 2011 (Turkey); 17 Hattula et al 2002 (USA)Saxena et al 2004 (India)

[illegible]

Section/Topic	Item No	Checklist item	19	20	21	22	23	24	25	26	27	28	29	30	31	32	33	34	35	36
**Title and abstract**																				
	1a	Identification as a randomised trial in the title	NR	✓	NR	✓	NR	NR	NR	NR	✓	NR	NR	NR	✓	✓	✓	✓	NR	NR
	1b	Structured summary of trial design, methods, results, and conclusions (for specific guidance see CONSORT for abstracts)	✓	P	✓	P	NR	NR	✓	✓	NR	✓	NR	NR		NR	✓	✓	NR	NR
**Introduction**																				
Background and objectives	2a	Scientific background and explanation of rationale	✓	✓	✓	✓	✓	✓	✓	✓	✓	✓	✓	✓	✓	✓	✓	✓	✓	✓
	2b	Specific objectives or hypotheses	✓	✓	✓	✓	✓	✓	✓	✓	✓	✓	✓	✓	✓	✓	✓	✓	✓	✓
**Methods**																				
Trial design	3a	Description of trial design (such as parallel, factorial) including allocation ratio	P	✓	✓	✓	✓	✓	✓	✓	✓	✓	✓	✓	✓	✓	✓	✓	✓	✓
	3b	Important changes to methods after trial commencement (such as eligibility criteria), with reasons	NR	NR	NR	NR	NR	NR	NR	NR	NR	NR	NR	NR	NR	NR	NR	NR	NR	NR
Participants	4a	Eligibility criteria for participants	✓	✓	✓	✓	✓	✓	✓	✓	✓	✓	✓	✓	✓	✓	✓	✓	✓	✓
	4b	Settings and locations where the data were collected	NR	NR	P	NR	NR	NR	NR	✓	NR	NR	NR	P	P	✓	✓	NR	✓	P
Interventions	5	The interventions for each group with sufficient details to allow replication, including how and when they were actually administered	✓	✓	✓	✓	✓	✓	✓	✓	✓	✓	✓	✓	✓	✓	✓	✓	✓	✓
Outcomes	6a	Completely defined pre-specified primary and secondary outcome measures, including how and when they were assessed	✓	✓	✓	✓	✓	✓	✓	✓	✓	✓	✓	✓	✓	✓	✓	✓	✓	✓
	6b	Any changes to trial outcomes after the trial commenced, with reasons	NR	NR	NR	NR	NR	NR	NR	NR	NR	NR	NR	NR	NR	NR	NR	NR	NR	NR
Sample size	7a	How sample size was determined	ü	✓	✓	✓	NR	NR	NR	NR	✓	NR	NR	NR	ü	✓	NR	✓	✓	NR
Harms	7b	When applicable, explanation of any interim analyses and stopping guidelines	NR	NR	NR	NR	NR	NR	NR	NR	NR	NR	NR	NR	NR	NR	NR	NR	NR	NR
**Randomisation**																				
Sequence generation	8a	Method used to generate the random allocation sequence	NR	✓	✓	✓	NR	✓	NR	✓	✓	NR	NA	NR	✓	NR	✓	✓	✓	✓
	8b	Type of randomisation; details of any restriction (such as blocking and block size)	NR	NR	✓	✓	NR	✓	NR	✓	✓	NR	NA	NR	✓	NR	NR	NR	NR	NR
Allocation concealment mechanism	9	Mechanism used to implement the random allocation sequence (such as sequentially numbered containers), describing any steps taken to conceal the sequence until interventions were assigned	NR	NR	NR	NR	NR	✓	NR	NR	✓	✓	NA	NR	✓	NR	NR	NR	NR	NR
Implementation	10	Who generated the random allocation sequence, who enrolled participants, and who assigned participants to interventions	NR	NR	NR	NR	NR	NR	NR	✓	NR	NR	NA	NR	✓	NR	NR	NR	P	NR
Blinding	11a	If done, who was blinded after assignment to interventions (for example, participants, care providers, those assessing outcomes) and how	NR	✓	✓	✓	✓	NR	✓	✓	✓	✓	NA	✓	✓	NR	NR	NR	✓	NR
	11b	If relevant, description of the similarity of interventions	NR	✓	NR	NR	NR	NR	NR	NR	NR	✓	NA	NR	P	✓	✓	✓	✓	✓
Statistical methods	12a	Statistical methods used to compare groups for primary and secondary outcomes	✓	✓	✓	✓	✓	✓	✓	✓	✓	✓	✓	✓	✓	✓	✓	✓	✓	✓
	12b	Methods for additional analyses, such as subgroup analyses and adjusted analyses	NA	NA	NA	NA	NA	NA	NA	NA	✓	NA	NA	NA	NA	NA	NA	NA	NA	✓

Section/Topic	Item No	Checklist item	19	20	21	22	23	24	25	26	27	28	29	30	31	32	33	34	35	36
**Results**																				
Participant flow (a diagram is strongly recommended)	13a	For each group, the numbers of participants who were randomly assigned, received intended treatment, and were analysed for the primary outcome	NR	NR	NR	NR	NR	NR	NR	NR	P	NR	NR	NR	NR	✓	✓	✓	NR	✓
	13b	For each group, losses and exclusions after randomisation, together with reasons	NR	P	✓	✓	✓	NR	✓	✓	✓	NR	NA	NR	✓	✓	✓	✓	NR	NR
Recruitment	14a	Dates defining the periods of recruitment and follow-up	NR	NR	✓	NR	NR	NR	NR	NR	NR	NR	NR	NR	✓	NR	✓	NR	✓	NR
	14b	Why the trial ended or was stopped	NR	NR	NR	NR	NR	NR	NR	NR	NR	NR	NA	NR	NR	NR	NR	NR	NR	NR
Baseline data	15	A table showing baseline demographic and clinical characteristics for each group	NR	✓	✓	✓	✓	✓	NR	✓	✓	NR	NR	NR	✓	✓	✓	NR	✓	✓
Numbers analysed	16	For each group, number of participants (denominator) included in each analysis and whether the analysis was by original assigned groups	P	P	P	P	✓	✓	✓	✓	✓	✓	P	P	✓	✓	✓	✓	✓	✓
Outcomes and estimation	17a	For each primary and secondary outcome, results for each group, and the estimated effect size and its precision (such as 95% confidence interval)	✓	P	✓	✓	✓	✓	✓	✓	✓	✓	P	✓	✓	✓	✓	✓	✓	✓
	17b	For binary outcomes, presentation of both absolute and relative effect sizes is recommended	NA	NA	NA	NA	NA	NA	NA	NA	NA	NA	NA	✓	P	NA	NA	NA	NA	NA
Ancillary analyses	18	Results of any other analyses performed, including subgroup analyses and adjusted analyses, distinguishing pre-specified from exploratory	NA	NA	NA	NA	NA	NA	NA	NA	✓	NA	NA	P	NA	NA	NA	NA	NA	✓
Harms	19	All important harms or unintended effects in each group (for specific guidance see CONSORT for harms)	NR	P	✓	✓	✓	NR	✓	✓	✓	NR	NR	✓	P	NR	✓	✓	NR	NR
**Discussion**																				
Limitations	20	Trial limitations, addressing sources of potential bias, imprecision, and, if relevant, multiplicity of analyses	NR	NR	✓	NR	NR	NR	P	NR	✓	NR	NR	NR	NR	P	P	P	✓	✓
Generalisability	21	Generalisability (internal validity, applicability) of the trial findings	✓	✓	✓	✓	✓	✓	✓	✓	✓	✓	✓	✓	✓	P	NR	P	✓	✓
Interpretation	22	Interpretation consistent with results, balancing benefits and harms, and considering other relevant evidence	✓	✓	✓	✓	✓	✓	✓	✓	✓	✓	✓	✓	✓	✓	✓	✓	✓	✓
**Other information**																				
Registration	23	Registration number and name of trial registry	NR	NR	NR	NR	NR	NR	NR	NR	NR	NR	NR	NR	NR	NR	NR	NR	NR	NR
Protocol	24	Where the full trial protocol can be accessed, if available	NR	NR	NR	NR	NR	NR	NR	NR	NR	NR	NR	NR	NR	NR	NR	NR	NR	NR
Funding	25	Sources of funding and other support (such as supply of drugs), role of funders	NR	NR	✓	NR	✓	NR	✓	✓	✓	✓	NR	NR	NR	NR	NR	NR	✓	NR

19. Steinbrook et al 1993 (USA); 20. Deshpande et al 2010 (India); 21. Agarwal et al 2007 (India); 22. Agarwal et al 2007 (India); 23.O'Connor et al 1995 (UK); 24. Smith et al 2002 (USA); 25. Ashburn et al 1997 (USA); 26 Vaghadia et al 1997 (Canada); 27. Kitamoto et al 1992 (Japan); 28. Maddi et al. 1990 (USA); 29.Brown et al. 1999 (USA); 30. Dickey et al. 1988 (USA); 31. Agarwal et al 2006 (India); 32. Ganter-Ritz et al. 2012 (USA); 33 Gupta et al. 2013 (India); 34 Yeoh et al. 2012 (Malaysia); 35 Deguzman et al. 2012 (USA); 36 Windle et al. 2006 (USA)

□, item present; NR not reported; P, partially complete; NA, not applicable
