# Supplementary material for: First do no harm: pain relief for the peripheral venous cannulation of adults, a systematic review and network meta-analysis
Source: BMC Anesthesiol. 2016 Oct 1;16:81. doi: 10.1186/s12871-016-0252-8 (PMC5045592; doi:10.1186/s12871-016-0252-8)
Supplement: Supplementary file 9 [file 12871_2016_252_MOESM6_ESM.docx]

Study year (location)	design	N	intervention control	dose	delivery	premed	outcome measure	median (IQR [range])	mean (SD [SEM])	mean difference (95%CI)[SE]
Agarwal et al. 2006 (India)	RCT	24	Diclofenac	100 mg	patch	NR	VAS 10 cm	3.0 (2.0-4.0)		
		24	Placebo	NA	patch			6.5 (4.5-7.0)		(Sig differences diclofenac vs placebo and diclofenac-buttock (p<0.05))
		24	Diclofenac - buttock	100 mg	patch			5.0 (4.3-7.8)		
Agarwal et al. 2007 (India)	RCT	150	Diclofenac	100 mg	patch	NR	VAS 10cm	0 (2)		
		150	EMLA and placebo patch	2 g	cream	NR		0 (1)		Sig differences EMLA vs placebo and diclofenac vs placebo (p=0.001)
		150	Placebo	NA	patch			6 (3)		
Agarwal et al. 2007 (India)	RCT	30	EMLA	NR	cream	2 x 2 mg lorazepam	VAS 10cm	1.5 (3)		
		30	Myolaxin	NR	ointment			1.5 (2)		(p=0.46)
Armstrong et al. 1990 (UK)	RCT	40	lidocaine 1%	0.2 ml	intradermal injection	No	VAS 10cm		0.7 (0.9)	lidocaine vs control: 3.1 (3.81, 2.39)
		40	Ethyl chloride	10 s	spray	No			1.8 (1.5)	Ethyl chloride vs control: 11 (3, 19)
		40	Control	NA	NA				3.8 (2.1)	
Ashburn et al. 1997 (USA)	RCT	21	Iontocaine	NR	Ionthophoresis (40 mA minutes)	NR	VAS 10 cm		0.7 (1.1)	1.4
		20	Saline	NR	sham ionthophoresis (40 mA minutes)				2.1 (2)	(2.39, 0.41)*
Beck et al 2011 (USA)	RCT	30	Bupivacaine 0.5%	0.2 mL	intradermal injection	NR	VAS 100mm	2.5 [0-12]		
		30	lidocaine 1% with NaHCO3 8.4% (9:1 ratio)	0.2 mL	intradermal injection			1 [0-59]		p=0.394
		30	lidocaine 1%	0.2 mL	intradermal injection			3.5 [0-26]		
		30	Chloroprocaine 2%	0.2 mL	intradermal injection			10[0-26]		
Biro & Cummins 1997 (Switzerland)	RCT	30	lidocaine 2%	0.3-0.5 ml	intracutaneuos injection	No	VAS 100 points	0 (0-10)	6.17 (9.62)	NR
		28	EMLA	2 g	cream			12.5 (0-25)	14.46 (13.90)	
		30	Ethyl Chloride	until a layer of frosting had developed	spray			25 (10-35)	26.17 (23.03)	(Sig differences ethyl chloride vs control (p<0.05))
		29	Placebo	2 g	cream			30 (20-60)	37.59 (25.55)	
		29	Control	NA	NA			30 (20-55)	34.83 (21.32)	

Study year (location)	design	N	intervention control	dose	delivery	premed	outcome measure	median (IQR [range])	mean (SD [SEM])	mean difference (95%CI)[SE]
Brown & Larson 1999 (USA)	Controlled trial	50	lidocaine 1%	0.1 ml	intradermal injection	NR	Visual numerical 0-10 & faces		1.4 (1.96)	lidocaine vs control 1.4
		50	Control	NA	NA				2.8 (2.49)	(2.29, 0.51)*
Brown 2003 (USA)	Survey	women:65 men: 14	lidocaine	NR	0	NA	Numeric 0-10		women:2.4 (2.1) men: 1.6 (1.74)	women: 0.87 (1.78, 0.038)*
		women:47 men: 54	Control	NA	NA				women:3.27 (2.75) men: 3.24 (2.7)	men: 1.64 (3.18, 0.10)*
Burke et al 2011 (USA)	RCT	73	lidocaine 1% with NaHCO3	0.2 ml	intradermal injection	NR	Verbal 0-10		1.56 (1.99)	1.02
		72	Saline 0.2ml	0.2 ml	intradermal injection				2.58 (2.50)	(1.761, 0.28)*
Campbell-Jones 2010 (USA)	RCT	20	lidocaine 1%	0.4 ml	intradermal injection	NR	Visual numerical 0-10		0.6 (1.187)	lidocaine vs saline: 2.29
		22	Saline with preservatives	0.4 ml	intradermal injection				0.6818 (0.945)	(3.53, 1.04)*
		18	Saline 0.4ml	0.4 ml	intradermal injection				2.8889 (2.447)	
Celik et al. 2011 (Turkey)	controlled cross-over	41	EMLA	2ml	cream	NR	VAS 100mm		10.7 (10.6)	18.1 (11.63, 24.57)*
		41	Ethyl chloride		spray				14.0 (12.4)	14.8 (8.03, 21.57)*
		41	Placebo cream		cream				33.4 (19.5)	-4.6 (-12.83, 3.63)*
		41	control		no treatment				28.8 (17.9)	
Deguzman et al. 2012 (USA)	RCT	192	Buffered lidocaine	0.1 to 0.2 ml	injection	No	Pain measured on a NRS (0 -10 scale).		1.19 (1.59)	0.53 (0.21, 0.85)*
		184	Bacteriostatic saline	0.1 to 0.2 ml	injection	No			1.72 (1.58)	
Deshpande et al 2010 (India)	RCT	100	EMLA all 60-70 min	2 g	cream	NR	VAS 100mm	8 (0-43)	9.449 (9.762)	Participant scores
		100	Diclofenac	100 mg	patch			25 (0-52)	23.79 (10.25)	14.341
										(17.132, 11.55)*
		100	EMLA	2 g	cream			10 (0-40)	9.584 (8.993)	Observer scores
		100	Diclofenac	100 mg	patch			25 (0-48)	24.44 (10.22)	14.856
										(17.541, 12.171)*

Study year (location)	design	N	intervention control	dose	delivery	premed	outcome measure	median (IQR [range])	mean (SD [SEM])	mean difference (95%CI)[SE]
Dickey 1988 (USA)	Controlled trial	11	lidocaine	0.1 ml	intradermal injection	No	VAS 100mm		9.63 (6.03)	lidocaine vs control: 23.22
		10	Saline 0.1ml	0.1 ml	intradermal injection				27.45 (12.47)	(37.93, 8.51)*
		10	Control	NA	NA				32.85 (22.49)	
Ganter-Ritz et al. 2012 (USA)	RCT	84	lidocaine 1%	NR	intradermal injection	NR	VAS 0-10		0.8 (1.0)	.6
		83	Saline with benzyl alchol	NR	intradermal injection	NR			1.4 (2.1)	(1.101623; .0983772)
Gunawardene 1990 (UK)	RCT	25	EMLA all 60 min	2.5 g	cream	No	Verbal 0-10	0 (0-0)	0.24 (0.66)	EMLA vs placebo: 5.08
		25	Placebo	NR	jelly			5 (5-5)	5.32 (0.62)	(5.44, 4.72)*
Gupta et al. 2013 (India)	RCT	30	EMLA	NR	cream	yes	incidence of no pain 0-10 VAS	1.5 (3)		0.04
		30	Myolaxin	NR	cream	yes		1.5 (3)		0.04
		30	EMLA + Myolaxin	NR	cream	yes		1.0 (2)		
		30	placebo	NR	cream	yes		3.0 (1)		<0.001
Harasawa 1993 (Japan)	RCT	12	lidocaine 50% 15 min	15 mg	patch	No	VAS 100mm		28.4 (13.1)	23.4
		14	lidocaine 50% 30 min	15 mg	patch				51.8 (15.9)	(35.3217, 11.4783)*
Hattula et al. 2002 (USA)	RCT	11	Buffered lidocaine 1%	0.1mL	intradermal injection	NR	VAS (range of scale not reported, assumed to be 0-10 on the basis of the results)		1.3(1.0)	buffered lidocaine vs.control 1.8(0.48, 3.12)
		13	Buffered saline 0.9% benzyl alcohol	0.2mL	intradermal injection	NR			2.7(1.4)	buffered saline vs. control 0.4 (-1.06,1.86)
		9	Control - no treatment			NR			3.1(1.9)	buffered lidocaine vs. buffered saline 1.4 (0.35, 2.45)
Hussey et al 1997 (USA)	RCT	33	lidocaine	0.1 ml	intradermal injection	No	VAS 100mm		1.4 (1.6)	lidocaine vs saline: 2.6
		33	Saline	0.1 ml						
		33	Control		NA				4 (2.5)	(3.61, 1.59)*

Study year (location)	design	N	intervention control	dose	delivery	premed	outcome measure	median (IQR [range])	mean (SD [SEM])	mean difference (95%CI)[SE]
Kitamoto et al 1992 (Japan)	RCT	16	lidocaine 10%	NR	gel	NR	Numeric 0-4		1 (0.5)	lidocaine vs placebo 1.3
	cross-over	16	Placebo	NR	gel				2.3 (0.3)	(1.6, 1.0)*
Lysakowski et al 2003 (USA)	RCT	89	lidocaine 1%	0.5 ml	dermal J-Tip needle-less	7.5 mg midazolam	Verbal 0-10		3.0 (2.8)	lidocaine 1% vs control: 0.9
		83	lidocaine 2%	0.5 ml					1.7 [1.7]	(1.81, 0.10)*
		89	saline						4.2 [2.5]	lidocaine 2% vs control: 2.2
		99	control					NR	3.9 (2.5)	(2.99, 1.41)*
Maddi et al 1990 USA	RCT	30	EMLA 5% 30 min	5 ml	cream	cardiac patients :	VAS 10cm	NR	NR	EMLA 30 min vs placebo: 0.07 (0.09)
	cross-over design	30	Placebo	5 ml	cream	0.1 mg/kg morphine sulphate and 0.4 mg scopolamine		NR	NR	
						orthopedic patients:				
		20	EMLA 45 min	5 ml	cream	10- 15 mg diazepam		NR	NR	EMLA 45 min vs placebo: 1.41 (2.45)
		20	Placebo	5 ml	cream			NR	NR	
		25	EMLA 60min	5 ml	cream			NR	NR	EMLA 60 min vs placebo: 1.08 (3.36)
		25	Placebo	5 ml	cream			NR	NR	
Miller & Balakrishnan 2001 (USA)	RCT	10	lidocaine 1%	0.3-0.5 ml	subcutaneus injection	NR	VAS 100mm		6.4 (8.5)	lidocaine vs EMLA:
		10	EMLA	2.5 g	cream				22.3 (20.1)	15.9
		10	Iontocaine (2% lidocaine & 1:100,000 epinephrine)	1 ml	Ionthophoresis (40 mA minutes)				4.2 (7.2)	(34.29, 2.49)*
										Iontocaine vs EMLA:
										18.1
										(36.27, 0.07)*
Molodecka et al 1994 a (UK)	RCT	30	EMLA 30min	2.5 g	cream	NR	VAS 100mm	19.0 (10.4) [1-81]		
		29	Amethocaine 5%	1 g	cream			18.0 (14.0) [0-82]		(no sig differences for 30 min (p=0.65) or 60 min (p=0.36) duration)
		30	EMLA 60min	2.5 g	cream			13.5 (10.5) [1-81]		
		29	Amethocaine 5%	1 g	cream			8.5 (9.6) [0-34]		

Study year (location)	design	N	intervention control	dose	delivery	premed	outcome measure	median (IQR [range])	mean (SD [SEM])	mean difference (95%CI)[SE]
Moppett 2004 (UK)	RCT	20	Iontocaine and placebo cream	3 ml	Ionthophoresis (20 mA min)	NR	Verbal 0-10	3 [0-6]		
	cross-over design	20	EMLA	NR	cream & sham ionthoporesis			1 [0-7]		p=0.023
O'Connor & Tomlinson 1995 (UK)	RCT	21	Amethocaine 4%	1 g	gel	No	VAS 100mm	7 [0-62]		
		21	Placebo	1 g	gel			27 [1-88]		p=0.01
Patterson et al 2000 (USA)	RCT	65	lidocaine 0.5 % with methylparaben	0.1 ml	subcutaneus injection	NR	VAS 100mm		1.3 [0.3]	lidocaine vs EMLA 0.88 (1.71, 0.049)*
		49	EMLA	2.5 g	cream				2.18 [0.3]	
		61	Saline with 0.9% benzyl alcohol	0.1 ml	subcutaneus injection				1.92 [0.3]	lidocaine vs saline 0.62 (1.4514, 0.2114)*
		63	Dichlorotetrafluoroethane	10 s	spray			NR	2.5 [0.3]	
Ravishankar et al 2012 (UK)	RCT	50	Ametop (4% tetracaine)	NR	gel	NR	VAS 100mm	11 (5-20 [0-72])		
		50	Rapydan (7% lidocaine and 7% tetracaine)	NA	patch			10 (5-24 [0-95])		
Saxena 2004 (India)	RCT	20	lidocaine 2%	2 g	gel	NR	VAS 100mm		36.1	NR
		20	EMLA	2 g	cream				7.7	NR
		20	Placebo	2 g	vaseline				45.25	
Selby & Bowles 1995 (UK)	RCT	40	lidocaine 1%	0.2 ml	subcutaneus injection	No	VAS 100mm	application pain 10.0 (3.5-19.0)		lidocaine 1% vs control: 13.5 (5, 21)
								PVC insertion pain 1.0 (0.0–10.5)		
		40	EMLA	0.2 ml	cream	No		application pain 0.0 (0.0-1.0)		
								PVC insertion pain 17.0 (6.0–26.0)		EMLA vs control: 23.5 (16, 30)
		40	Ethyl chloride	10 s	spray	No		application pain 9.5 (3.0-21.5)		
								PVC insertion pain 4.0 (0.0–21.0)		Ethyl chloride vs control: 14 (3, 19)
		40	Control	NA	NA			PVC insertion pain 23.5 (12.0–47.75)		
Smith et al 2002 (USA)	Controlled trial	20	EMLA all 5 min	2.5 g	cream	No	Verbal 0-10	3 (2-4)	3.25 (1.10)	EMLA vs placebo: 2.05
		20	Placebo	2.5 g	cream			6 (4.5-7)	5.3 (1.87)	(3.29, 0.81)*

Study year (location)	design	N	intervention control	dose	delivery	premed	outcome measure	median (IQR [range])	mean (SD [SEM])	mean difference (95%CI)[SE]
Steinbrook et al 1993 (USA)	RCT	95	lidocaine 1%	0.1-0.2 ml	subcutaneus injection	No	VAS 100mm	4 [0–86]	12 (16) [2]	lidocaine vs lidocaine + NaCO3 3
		89	lidocaine 1% with NaCO3	0.1-0.2 ml	subcutaneus injection			3 [0–69]	9 (13) [1]	(1.26, 7.26)*
Vaghadia et al. 1997 (Canada)	RCT	24	EMLA	1 g	patch	No	VAS 100mm	8 [0-92]		
		26	Placebo all 60- 90 min	1 g	patch			25 [0-98]		(Sig differences for participant (p<0.01) and observer (p<0.05) scores)
Windle et al. 2006	RCT	67	Lidocaine 1%	NR	subcutaneus injection	NR	VAS 100mm		8.16 (14.86)	19.31 (13.03, 25.59)
		58	Bacterio static normal saline with benzyl alcohol 0.9%	NR	subcutaneus injection	NR			13.61 (15.67)	13.86 (7.15, 20.57)
		60	No treatment	NR		NR			27.47 (20.71)	
Yeoh & Lee 2012 (Malaysia)	RCT	39	EMLA 5%	1ml	cream	No	VAS 100mm	30	27.9 (9.8)	NS
		41	Amethocaine 4%	1ml	cream	No		20	19.1 (14.1)	

* calculated from values presented in the paper
