# Supplementary material for: First do no harm: pain relief for the peripheral venous cannulation of adults, a systematic review and network meta-analysis
Source: BMC Anesthesiol. 2016 Oct 1;16:81. doi: 10.1186/s12871-016-0252-8 (PMC5045592; doi:10.1186/s12871-016-0252-8)
Supplement: Supplementary file 10 [file 12871_2016_252_MOESM9_ESM.docx]

Study year (location)	N	intervention	dose	delivery	premed	outcome measure	median (SD [SEM]) [range]	median difference (95%CI)[SE]	median (SD [SEM]) [range]	median difference (95%CI)[SE]
							LA application pain		cannulation pain	
Ganter-Ritz et al. 2012 (USA)	84	lidocaine 1%	0.1 mL	intradermal injection	NR	VAS	mean (SD) 2.2 (1.7)		mean (SD) 0.8 (1.0)	
RCT	85	Buffered lidocaine 1%	0.1 mL	intradermal injection		0-100	mean (SD) 0.9 (1.2)		mean (SD) 0.8 (1.1)	
	83	Buffered saline (benzyl alcohol)	0.1 mL	intradermal injection			mean (SD) 1.0 (1.2)		mean (SD) 1.4 (2.1)	
Beck et al 2011 (USA)	30	Bupivacaine 0.5%	0.2 mL	intradermal injection	NR	VAS	8.5 [1-54]	NS	2.2 [0-12]	NS
RCT	30	lidocaine 1% with NaHCO3 8.4% (9:1 ratio)	0.2 mL	intradermal injection		100mm	6 [0-65]	NS	1 [0-59]	NS
	30	lidocaine 1%	0.2 mL	intradermal injection			11 [2-52]	NS	3.5 [0-26]	NS
Windle et al. 2006	67	Lidocaine 1%	NR	subcutaneus injection	NR	VAS 100mm	16.94 (16.58)	5.79 (11.32, 0.26)*	8.16 (14.86)	lidocaine vs. control 19.31 (13.03, 25.59)*
RCT	58	Bacterio static normal saline with benzyl alcohol 0.9%	NR	subcutaneus injection	NR		11.15 (14.36)		13.61 (15.67)	saline vs. control 13.86 (7.15, 20.57)*
	60	No treatment	NR		NR				27.47 (20.71)	
	30	Chloroprocaine 2%	0.2 mL	intradermal injection			4.5 [0-30]	NS	1 [0-26]	NS
Patterson et al. 2000 (USA)	65	lidocaine 0.5 % with methylparaben	0.1 ml	subcutaneous injection	NR	VAS	1.08 [0.1]	lidocaine vs. saline:	1.3 [0.3]	lidocaine vs. EMLA -0.88
RCT	49	EMLA	2.5 g	cream		100mm	0 [0]	0.56	2.18 [0.3]	[-7.74, -0.02]*
	61	Saline with 0.9% benzyl alcohol	0.1 ml	subcutaneous injection			0.52 [0.1]	[0.28, 0.84]***	1.92 [0.3]	lidocaine vs. saline -0.62
	63	Dichlorotetrafluoroethane	10 s	spray			1.7 [0.1]	lido vs. DCTF	2.5 [0.3]	[-1.46, 0.22]
								0.62		lidocaine vs. dichlorotetrafluoroethane -1.20
								[-0.90, -0.34]***		[-2.40, -0.36]
Selby et al 1995 (UK)	40	lidocaine 1%	0.2 ml	subcutaneous injection	No	VAS	10.0 [3.5-19.0]		1.0 (0.0–10.5)	lidocaine vs. control -13.5 (-5, -21)
RCT	40	Control	NA	NA		100mm	-		23.5 (12.0–47.75)	
	40	EMLA	0.2 ml	cream			0.0 [0.0-1.0]		17.0 (6.0–26.0)	
	40	Ethyl chloride	10 s	spray			9.5 [3.0-21.5]		4.0 (0.0–21.0)	
Steinbrook et al 1993 (USA)	95	lidocaine 1%	0.1-0.2 ml	subcutaneous injection	No	VAS	12 [11]	lidocaine vs. lidocaine + NaHCO3]	12 [16]	lidocaine vs. lidocaine + NaHCO3]
RCT	89	lidocaine 1% with NaHCO3	0.1-0.2 ml	subcutaneous injection		100mm	8 [10]	*4 [0.94, 7.06]**	9 [13]	*3.00 [-1.26, 7.26]

* calculated from values presented in the paper, **p<0.05, ***p<0.0001, NS: not significant
